# Intra-articular sprouting of nociceptors accompanies progressive osteoarthritis: comparative evidence in four murine models

**DOI:** 10.3389/fnana.2024.1429124

**Published:** 2024-07-15

**Authors:** Alia M. Obeidat, Shingo Ishihara, Jun Li, Natalie S. Adamczyk, Lindsey Lammlin, Lucas Junginger, Tristan Maerz, Richard J. Miller, Rachel E. Miller, Anne-Marie Malfait

**Affiliations:** ^1^Division of Rheumatology, Department of Internal Medicine, Rush University Medical Center, Chicago, IL, United States; ^2^Department of Orthopaedic Surgery, University of Michigan, Ann Arbor, MI, United States; ^3^Department of Pharmacology, Northwestern University Feinberg School of Medicine, Chicago, IL, United States

**Keywords:** osteoarthritis, neuroplasticity, sprouting, nociceptive innervation, mouse models

## Abstract

**Objective:**

Knee joints are densely innervated by nociceptors. In human knees and rodent models, sprouting of nociceptors has been reported in late-stage osteoarthritis (OA). Here, we sought to describe progressive nociceptor remodeling in early and late-stage OA, using four distinct experimental mouse models.

**Methods:**

Sham surgery, destabilization of the medial meniscus (DMM), partial meniscectomy (PMX), or non-invasive anterior cruciate ligament rupture (ACLR) was performed in the right knee of 10-12-week old male C57BL/6 Na_V_1.8-tdTomato mice. Mice were euthanized (1) 4, 8 or 16 weeks after DMM or sham surgery; (2) 4 or 12 weeks after PMX or sham; (3) 1 or 4 weeks after ACLR injury or sham. Additionally, a cohort of naïve male wildtype mice was evaluated at age 6 and 24 months. Mid-joint cryosections were assessed qualitatively and quantitatively for Na_V_1.8+ or PGP9.5+ innervation. Cartilage damage, synovitis, and osteophytes were assessed.

**Results:**

Progressive OA developed in the medial compartment after DMM, PMX, and ACLR. Synovitis and associated neo-innervation of the synovium by nociceptors peaked in early-stage OA. In the subchondral bone, channels containing sprouting nociceptors appeared early, and progressed with worsening joint damage. Two-year old mice developed primary OA in the medial and the lateral compartment, accompanied by nociceptor sprouting in the synovium and the subchondral bone. All four models showed increased nerve signal in osteophytes.

**Conclusion:**

These findings suggest that anatomical neuroplasticity of nociceptors is intrinsic to OA pathology. The detailed description of innervation of the OA joint and its relationship to joint damage might help in understanding OA pain.

## Introduction

Chronic pain affects 20% of adults in the United States, causing tremendous suffering and a high economic burden (Steinmetz et al., 2023). The musculoskeletal system is one of the major sources of chronic pain, in particular low back pain and osteoarthritis (OA). OA is the most prevalent form of arthritis affecting synovial joints, and is characterized by progressive joint damage, ultimately leading to loss of joint function (Malfait and Block, 2015). Approximately 240 million individuals worldwide live with symptomatic OA (Allen et al., 2022; Lezin and Watkins-Castillo, 2022), with chronic pain as the main symptom (Bedson et al., 2007; Neogi, 2013). Available pain management options include non-steroidal anti-inflammatory drugs (NSAIDs), duloxetine, and intra-articular steroids (Bannuru et al., 2019), but these fall short of patients’ needs and their chronic use is often not safe, particularly in an older population (Malfait and Schnitzer, 2013). Furthermore, the wide availability of opioids has contributed to the ongoing opioid crisis (Deveza et al., 2018). Hence, OA pain is a formidable problem for which safe and effective pharmacological treatments are urgently needed. This pressing medical need has resulted in an increasingly intense focus on research aimed at unraveling mechanisms underlying pain in OA (Vincent, 2020; Malfait et al., 2021).

A critical gap hindering the development of effective OA pain therapeutics is our incomplete understanding of the nociceptive innervation of the joint. The healthy knee joint is supplied by a complex system of sympathetic and sensory neurons, predominantly nociceptors (Schaible and Grubb, 1993; Heppelmann, 1997; McDougall, 2006). Nociceptors, mostly unmyelinated C-fibers, innervate all joint tissues except for cartilage, which is aneural. Few studies have attempted to systematically describe the precise anatomical location of nociceptor nerve endings in the knee (Heppelmann, 1997; McDougall, 2006). OA is a disease of the whole joint, and all joint tissues undergo marked cellular and molecular changes as the disease progresses (Loeser et al., 2012). A major observation emerging from recent research in human and rodent OA knees is that the sensory innervation of the knee undergoes profound alterations during pathology. For example, in human osteoarthritic knee joints and in rodent models, calcitonin gene related peptide (CGRP)-immunopositive neurons have been described in osteochondral channels in the subchondral bone (Aso et al., 2020, 2022; Ishihara et al., 2021). Similarly, in late-stage experimental OA induced by surgical destabilization of the medial meniscus (DMM), nociceptor sprouting occurs in specific locations in the medial compartment, including synovium, meniscus, and within subchondral bone channels (Obeidat et al., 2019). Rodent models of OA provide a critical opportunity for studying the relationship between progressive joint damage, pain, and their underlying mechanisms (Malfait et al., 2013). In mice, knee OA can either be surgically induced by creating an instability, for example by DMM (Glasson et al., 2007) or through a partial meniscectomy (PMX) (Knights et al., 2012), as well as in a non-invasive manner by causing an anterior cruciate ligament rupture (ACLR) to model post-traumatic OA (PTOA) (Rzeczycki et al., 2021). C57BL/6 mice, especially males, also develop primary knee OA with age (Loeser, 2013; Geraghty et al., 2023). Joint pathology has been well described all in these models, and in all four models, we and others have extensively documented pain-related behaviors as disease progresses (Knights et al., 2012; Miller and Malfait, 2017; von Loga et al., 2020; Bergman et al., 2023; Geraghty et al., 2023). However, information on the precise anatomical distribution of nociceptive nerve endings in experimental knee OA, and the changes that occur in specific joint tissues as disease progresses, remains very limited. We propose that a precise description of the anatomical distribution of intra-articular nociceptors across several models is necessary as a prelude to our understanding of knee pain. Therefore, we sought to provide a detailed, longitudinal description of the nociceptive innervation of the mouse knee in these four widely used murine OA models (DMM, PMX, ACLR, and age-associated OA). These models all progress differently, and for each model, we selected timepoints spanning both early and later stages of disease severity as assessed by histological scoring of joint damage. We describe the precise distribution of intra-articular nociceptor endings as it relates to pathological changes in the joint tissues.

## Materials and methods

### Animals

**Figure 1:**
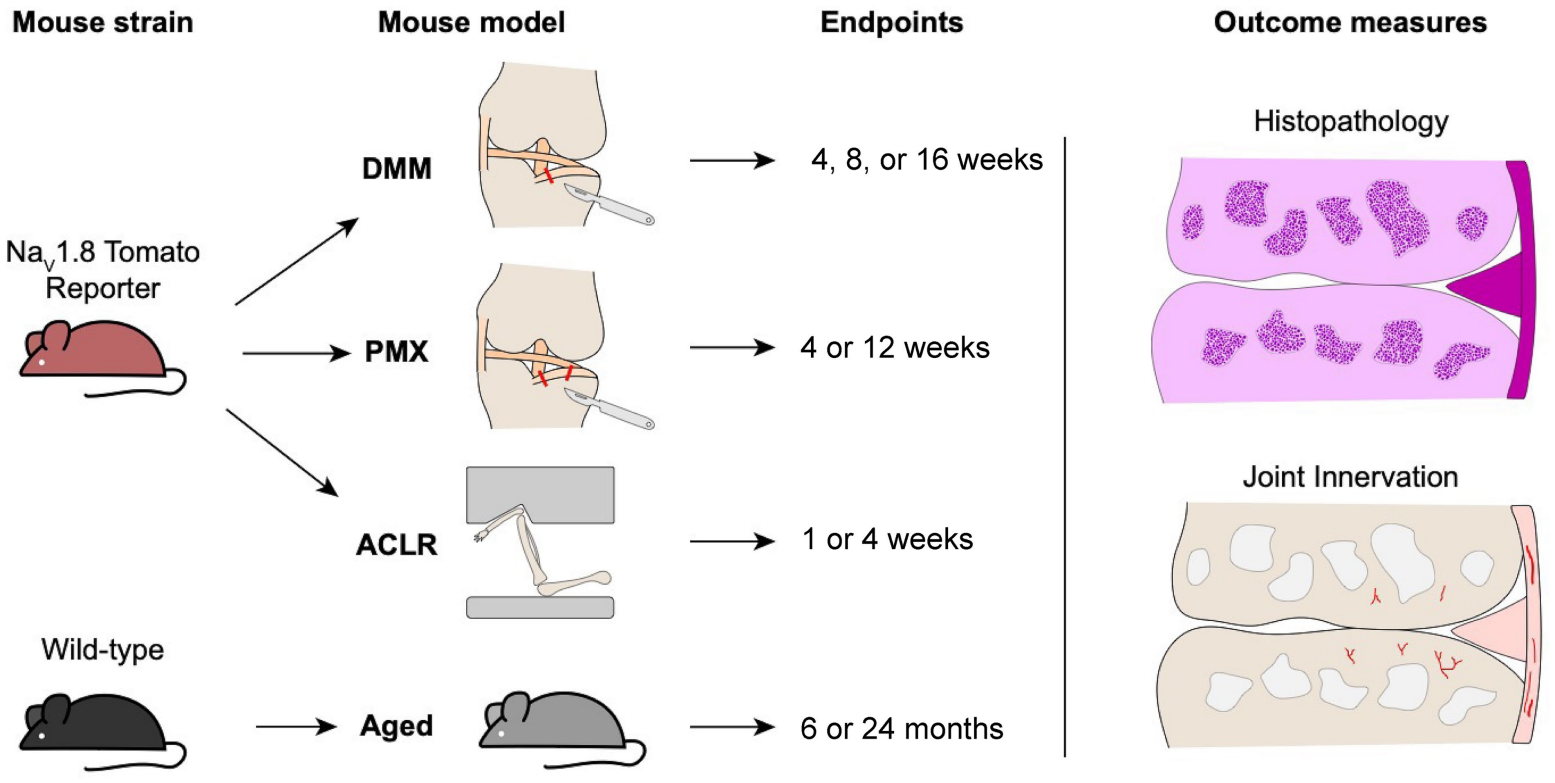
Schematic overview of male mouse strains, osteoarthritis models, timepoints, and outcome measures used in the study.

All experiments were performed in male mice C57BL/6 (*n* = 97), either wild-type (WT) mice (inbred at Rush) or Na_V_1.8Cre-tdTomato reporter mice. Na_V_1.8 is a marker for >90% of C-fibers, as well as a fraction of Aδ-nociceptors (Stirling et al., 2005; Shields et al., 2012), and we have previously shown a high extent of co-localization of Na_V_1.8+ with PGP9.5+ staining as well as the pan-DRG neuron marker, Pirt-GCaMP3 (Kim et al., 2016). All animals were bred in-house, and randomly assigned to an OA model and groups. Animals were housed with food and water *ad libitum* and kept on 12-h light cycles. Animal procedures were approved by the Institutional Animal Care and Use Committee at Rush University Medical Center or the University of Michigan. Figure 1 shows an overview of the study design.

### Destabilization of the medial meniscus (DMM)

DMM (*n* = 15) or sham surgery (*n* = 10) was performed in the right knee of 10-week old male Na_V_1.8-tdTomato reporter mice (Glasson et al., 2007). All DMM and sham surgeries were performed by the same surgeon (SI). Mice were sacrificed 4, 8 or 16 weeks after surgery (*n* = 5 per group). Please see details in Supplementary methods.

### Partial meniscectomy (PMX)

PMX (*n* = 10) or sham surgery (*n* = 10) was performed in the right knee of 10-week old male Na_V_1.8-tdTomato mice, as described (Knights et al., 2012). All PMX and sham surgeries were performed by the same surgeon (JL). Mice were sacrificed 4 or 12 weeks after surgery (*n* = 5 per group). Details in Supplementary methods.

### Anterior cruciate ligament rupture (ACLR)

At the age of 12–14 weeks, male Na_V_1.8-tdTomato reporter mice were randomized to a Sham group (*n* = 5) (anesthesia and analgesia only, no injury loading) or tibial compression-based, noninvasive ACLR (*n* = 9), using computer-aided block randomization. Mice were sacrificed at 1 week (PTOA synovitis) or 4 weeks (established PTOA) (Bergman et al., 2023). Details in Supplementary methods.

### Age-associated primary OA

Male WT C57BL/6 mice were aged without any interventions and sacrificed at age 26 weeks (*n* = 5) or 2 years (*n* = 5).

### Knee histology and immunohistochemistry

Twenty μm-thick coronal sections were collected from the mid-joint area defined previously in (Obeidat et al., 2019). Knee sections from WT mice used for the aging study were stained for the pan-neuronal marker, protein gene product 9.5 (PGP9.5), as described (Obeidat et al., 2019). Sections were imaged using a laser-scanning confocal microscope (Olympus IX70) and processed using ImageJ and Fluoview software (FV10-ASW 4.2 Viewer). Adjustments were made to brightness and contrast to reflect true colors (Obeidat et al., 2019). All images were treated the same in terms of adjustments to brightness and contrast to minimize bias. Details in Supplementary methods.

### Quantification of the neuronal signal per region

All quantifications were performed by an observer blinded to the treatment groups.

In the medial and lateral synovium, Na_V_1.8+ and PGP9.5+ signals were quantified in mid-joint coronal sections using ImageJ. Briefly, regions of interest (ROI) were manually outlined for each section using anatomical landmarks. Thresholds were adjusted for all images similarly to control for background. The area of positive signal within each ROI was measured and was normalized to the total area of the ROI; the percentage of positive signal per ROI is reported (Supplementary Figure S1).

Na_V_1.8+ and PGP9.5+ subchondral bone channels were quantified as previously described (Obeidat et al., 2019). The length of positive channels was measured using the ImageJ neuroanatomy plugin (Arshadi et al., 2021). The distance from the tidemark was measured using ImageJ and the shortest distance between the distal end of the channel and the tidemark was reported, and compared to baseline. The baseline was determined by averaging the distance for all existing Na_V_1.8 and PGP9.5 positive channels in naïve and sham control knees (since, while most of the age-matched sham and naïve controls lack these channels, there were some present). The number of branching points was counted for each mouse using a knee section with maximum branching (Supplementary Figure S2). Details in Supplementary methods.

### Knee histopathology

Knee sections from all groups were stained with hematoxylin (1x-Sigma HHS160) and eosin (1%-Sigma E4382) (H&E). Knee sections were evaluated for cartilage degeneration using a modified OARSI score, as described (Obeidat et al., 2019). For osteophyte scoring, one section with the major osteophyte was used to assess osteophyte width and maturity, as described (Little et al., 2009; Geraghty et al., 2023). Osteophyte measurements were performed using Osteomeasure software (OsteoMetrics) (Sekiya et al., 2022; Geraghty et al., 2023). Synovial hyperplasia, cellularity and fibrosis were evaluated as described (Geraghty et al., 2023; Obeidat et al., 2024). All scores were performed by an observer blinded to treatment groups. For further details, see Supplementary methods.

### Statistical analysis

Sample size was determined based on our previous data comparing innervation changes between sham and DMM (Obeidat et al., 2019). Ordinary two-way ANOVA was used for comparison of multiple groups and time points followed by Tukey’s multiple comparison test. For aging mice, unpaired two-tailed Student’s t test was used for pairwise comparisons. For comparison of branching between two timepoints, a Mann Whitney test was used. Statistical analyses were performed using GraphPad Prism 9. Data are presented as mean ± 95% CI. We performed correlation analyses between medial subchondral bone Na_V_1.8+ or PGP9.5+ channels and cartilage damage, and between medial synovium Na_V_1.8+ or PGP9.5+ fibers and medial synovitis scores using Prism 10. The correlation included 39 pair-wise comparisons from all OA groups (DMM *n* = 15, PMX *n* = 10, ACLR *n* = 9, aging *n* = 5).

## Results

### Knee histopathology

We first performed a detailed histopathological analysis of the knee joints in the four models, assessing OARSI score, synovitis, and osteophyte width and maturity at different stages of disease. We confirmed that in all models, mice developed cartilage degeneration (Supplementary Figures S3A,D,G,J), synovial pathology (Supplementary Figures S3B,E,H,K, S5), and osteophytes (Supplementary Figures S3C,F,I,L). Representative histological images of the medial side are shown in Supplementary Figure S4. Representative images of the whole joint are shown in Supplementary Figure S6, in addition to total cartilage degeneration scores and total synovial scores. Details in Supplementary results.

### Nociceptor sprouting

Having confirmed that mice developed progressive osteoarthritic changes in all four models, in concordance with published findings (Little et al., 2009; Gilbert et al., 2018; von Loga et al., 2020; Geraghty et al., 2023), we documented the distribution of free nerve endings in different joint tissues at all timepoints.

#### Nociceptors sprout in the synovium

**Figure 2:**
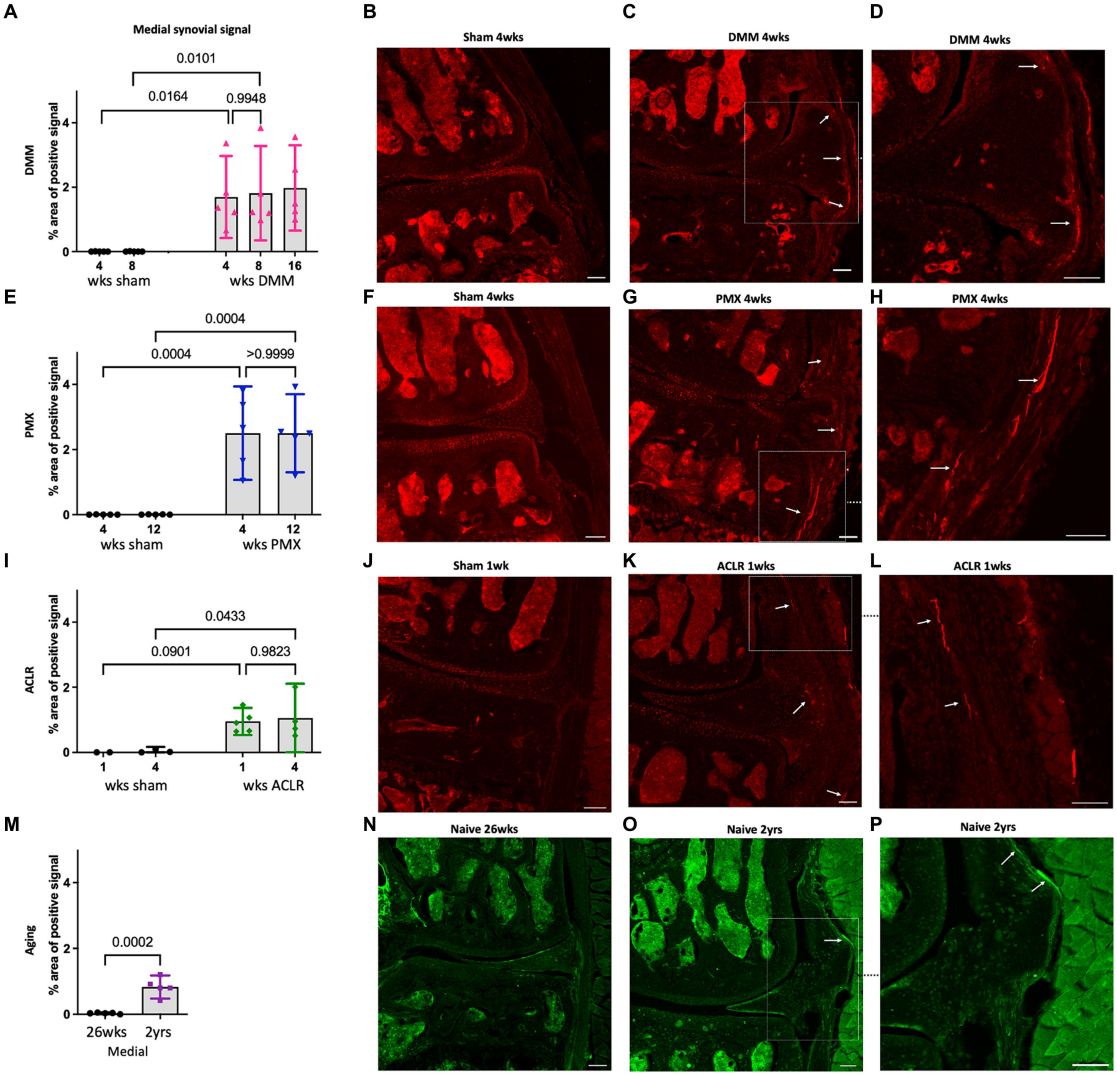
**(A)** Quantification of neuronal signal in the medial synovium 4, 8 and 16 weeks after sham and DMM surgeries (*n* = 5/group); **(B–D)** Representative confocal images of Na_V_1.8-tdTomato mouse knees showing the nerve fibers in the medial synovium (white arrows) at 4 weeks after sham and DMM surgery, with **(D)** showing a magnified image of **(C)**; **(E)** Quantification of neuronal signal in the medial synovium 4 and 12 weeks after sham and PMX surgeries (*n* = 5/group); **(F–H)** 4 weeks after sham, 4 weeks after PMX, and zoomed in image, respectively; **(I)** Quantification of neuronal signal in the medial synovium 1 and 4 weeks after sham and ACLR injury (*n* = 4–5/group); **(J–L)** 1 week after sham, 1 week after ACLR injury, and zoomed in image, respectively; **(M)** Quantification of neuronal signal in the medial synovium of 26-week old and 2-year old naïve mice (*n* = 5/group); **(N–P)** PGP9.5 staining in a 26-week old mouse, a 2-year old mouse, and zoomed in image, respectively. Mean ± 95% CI. Scale bar = 100 μm.

We have previously reported profound neuroplasticity of intra-articular nociceptors in the medial compartment of the murine knee joint 16 weeks after DMM, where we observed sprouting of nociceptors in the medial synovium (Obeidat et al., 2019). Here, we assessed Na_V_1.8-innervation at earlier timepoints after DMM vs. sham surgery, and found that 4 and 8 weeks after DMM, innervation of the deeper sublining layers of the medial synovium was increased compared to sham controls (Figures 2A–D). This occurred as early as 4 weeks after DMM, at which time synovitis peaked (Supplementary Figure S3B), with no further increase observed by weeks 8 and 16 (Figure 2A). Representative sections at week 4 are shown in (Figures 2B–D), and synovial innervation 8 and 16 weeks after DMM in Supplementary Figures S7A,B,E,F. In the lateral synovium, Na_V_1.8-innervation decreased with age in both the DMM and sham groups starting at the 8-week timepoint (i.e., 18 weeks of age), and no further decrease was observed at week 16 (Supplementary Figure S8A). This age-related decrease of nociceptor density in the lateral synovium is compatible with our previously reported findings (Obeidat et al., 2019). No neo-innervation was observed in the lateral synovium at any time, concordant with the lack of synovitis or OA changes in the lateral knee compartment.

We then assessed the nociceptive innervation of the synovium in three additional models. For the PMX model, we selected week 4 as early disease and week 12 as late-stage disease, based on reports in the literature, and confirmed by the histological findings described above. Four weeks after PMX, moderate cartilage damage (Supplementary Figure S3D) was accompanied by synovitis (Supplementary Figures S3E, S5D–F), and an increased Na_V_1.8 signal in the superficial and deep layers of the medial synovium. No further innervation changes were detected by week 12 *post* PMX (Figures 2E–H). Representative sections at week 4 are shown in Figures 2F–H, and synovial innervation at 12-weeks *post* PMX in Supplementary Figures S7G,H. The innervation of the lateral synovium in the PMX model also declined with age in both sham and PMX groups by week 12, with no evidence of new innervation (Supplementary Figure S8D).

We selected the ACLR model as a non-invasive injury model of PTOA and assessed joint innervation 1 and 4 weeks after injury. The early 1-week timepoint was characterized by mild cartilage degeneration (Supplementary Figure S3G), robust synovitis (Supplementary Figures S3H, S5G–I), and fine Na_V_1.8 fibers had already sprouted into the deep and superficial layers of the medial synovium (Figures 2I–L). No further changes in the medial synovial innervation were detected 4 weeks after injury compared to the early timepoint (Figure 2I and Supplementary Figures S7K,L), despite the development of severe cartilage degeneration by this timepoint, especially at the femoral condyles. The innervation in the lateral synovium did not change between weeks 1 and 4 in either group (Supplementary Figure S8G).

Finally, immunostaining for PGP9.5 was used to evaluate neuronal sprouting in knees of naïve male C57BL/6 mice at the ages of 6 and 24 months. Six-month old mice showed no nociceptor sprouting in the synovium, concordant with the absence of OA degenerative changes (Supplementary Figures S3J–L). By 2 years of age, mild joint damage (as observed in Supplementary Figure S3J) and synovial changes were accompanied by an increase in the sensory innervation in the deep layers of the medial synovium (Figures 2M–P). No changes were observed in innervation of the lateral synovium between 6 and 24 months (Supplementary Figure S8J).

#### Nociceptors sprout in subchondral bone channels and within osteophytes

**Figure 3:**
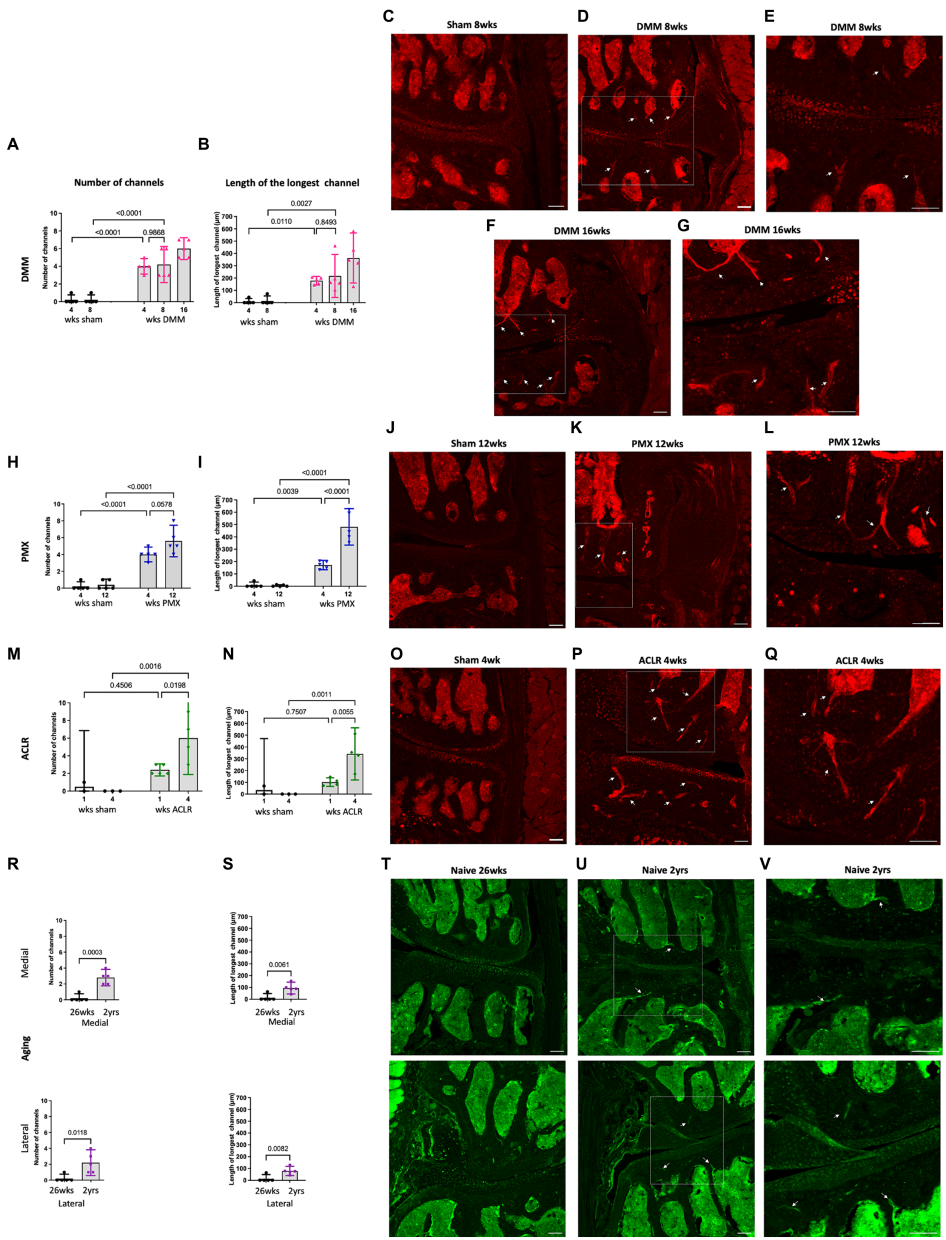
Quantification of neuronal signal in the medial subchondral bone 4, 8 and 16 weeks after sham and DMM surgeries showing **(A)** the number of Na_V_1.8 + channels; **(B)** the length of the longest channel; **(C–E)** Representative confocal images of Na_V_1.8-tdTomato mouse knees showing fibers within medial subchondral bone channels (white arrows) at 8 weeks after sham and 8 and 16 weeks after DMM surgery; **(E,G)** magnified image of medial subchondral bone fibers at 8 and 16 weeks after DMM; **(H,I)** quantification of the number of positive channels and the length of the longest channel, respectively, at 4 and 12 weeks after sham and PMX surgeries; **(J–L)** 4 weeks after sham, 4 weeks after PMX surgery, and zoomed in image, respectively; **(M,N)** quantification of the number of positive channels and the length of the longest channel, respectively; **(O–Q)** 1 week after sham, 1 week after ACLR injury, and zoomed in image, respectively; **(R,S)** quantification of the number of positive channels and the length of the longest channel, respectively, in 26-week old and 2-year old naïve mice in the medial and lateral compartment; **(T–V)** PGP9.5 staining in the medial and lateral compartment of 26-week old mice, 2-year old naïve mice, and zoomed in image, respectively. Mean ± 95% CI. Scale bar = 100 μm.

**Figure 4:**
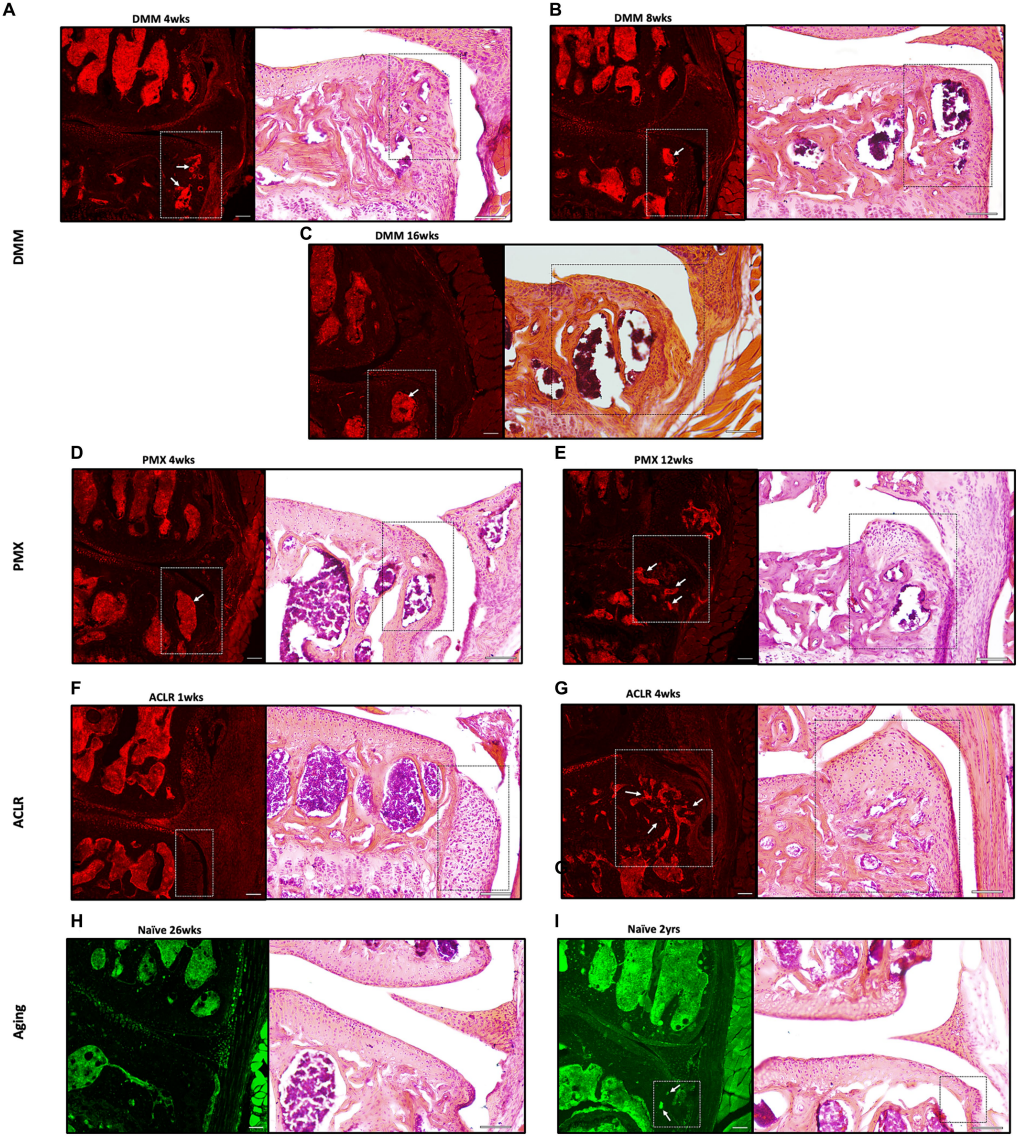
Representative confocal and H&E stained images of Na_V_1.8-tdTomato and PGP9.5-stained WT knees showing neuronal signal within osteophytes (white and black insets respectively) at **(A–C)** 4,8 and 16 weeks after DMM; **(D,E)** 4 and 12 weeks after PMX; **(F,G)** 1 and 4 weeks after ACLR; **(H,I)** 26-week old and 2-year old naïve mice. White arrows indicate Na_V_1.8+ signal within the bone marrow of the osteophytes. Scale bar = 100 μm.

We previously reported the presence of Na_V_1.8+ nociceptors in channels in the sclerotic subchondral bone, 16 weeks after DMM (Obeidat et al., 2019). Here, we analyzed additional timepoints after DMM, and counted the number of medial and lateral channels with a nociceptor signal, as well as the length of the longest channel. This revealed sprouting of Na_V_1.8+ nerve fibers within medial subchondral bone channels, 4, 8 and 16 weeks after DMM. We detected no difference in the number or length of channels between the 2 early timepoints (4 and 8 weeks) (Figures 3A,B). However, the number of Na_V_1.8 positive channels increased 16 weeks after DMM, as well as the length of the longest channel (Figures 3A,B) (number of channels DMM 4 vs. 16 weeks *p* = 0.09, length DMM 4 vs. 16 weeks *p* = 0.07, one-way ANOVA). Representative images of these channels at 8 and 16 weeks after DMM are shown in Figures 3C–G. We also assessed the distance from the tidemark and the number of branching points in the nerves within the channels. Na_V_1.8+ channels were closer to the tidemark and more branched 16 weeks *post* DMM compared to the earlier timepoints (Supplementary Figures S9A,I) (distance from the tidemark DMM 4 vs. 16 weeks *p* = 0.027, one-way ANOVA). Representative images at week 4 are shown in Supplementary Figures S7C,D. Lateral subchondral bone channels were not different between DMM and shams in any of the parameters measured (Supplementary Figures S8B,C, S9E). In addition to sprouting in subchondral bone channels, we also detected Na_V_1.8+ signal within osteophytes at all timepoints *post*-DMM (Figures 4A–C).

Similar to DMM, PMX-operated mice showed a significant increase in number of positive channels compared to age-matched sham controls (Figure 3H). These channels were prominent by week 4, and by week 12 *post*-PMX, Na_V_1.8+ channels appeared to increase in number (Figure 3H), were longer (Figure 3I), closer to the tidemark (Supplementary Figure S9B), and more branched (Supplementary Figure S9J), compared to week 4. Here too, lateral subchondral bone channels were not different between PMX and age-matched shams in any of the parameters measured (Supplementary Figures S8E,F, S9F). Representative images showing Na_V_1.8+ subchondral bone channels at 12 weeks *post*-PMX are shown in Figures 3J–L, and at 4 weeks *post*-PMX in Supplementary Figures S7I,J. We also detected a strong Na_V_1.8+ signal within osteophytes at both timepoints after PMX (Figures 4D,E).

One week after ACLR, the subchondral bone showed mild changes, with few Nav1.8+ fibers detectable within channels (Supplementary Figures S7M,N). By 4 weeks *post-*injury, however, Na_V_1.8+ subchondral bone channels were significantly greater in number (Figure 3M), longer (Figure 3N), and closer to the tidemark (Supplementary Figure S9C) compared to shams and to the early timepoint. No difference in number and length of lateral channels was observed between ACLR and sham groups (Supplementary Figures S8H,I). Representative images at 4 weeks are shown in Figures 3O–Q, and at 1 week after ACLR in Supplementary Figures S7K,L. No branching was observed at either timepoint (Supplementary Figure S9K). While the chondrophytes at the 1-week timepoint did not contain nociceptors (Figure 4F), mature osteophytes showed Na_V_1.8+ signal 4 weeks *post* ACLR (Figure 4G).

Finally, PGP9.5 staining of naive knees showed no subchondral bone sprouting in 26-week old mice, when there were no signs yet of primary OA. By age 2 years, joint damage in both the medial and lateral compartments was accompanied by innervation changes, with PGP9.5+ channels present in subchondral bone in both compartments (Figures 3T–V). These channels were significantly greater in number and longer compared to the younger knees (Figures 3R,S). Channels were closer to the tidemark compared to controls (Supplementary Figures S9D,H). No branching was detected in either compartment. Innervation changes were more pronounced in the medial than in the lateral compartment, reflecting worse damage in the medial compartment. While no osteophytes were observed in 26-week old mice (Figure 4H), in some 2-year old mice, a PGP9.5+ signal was detected within small osteophytes (Figure 4I).

**Figure 5:**
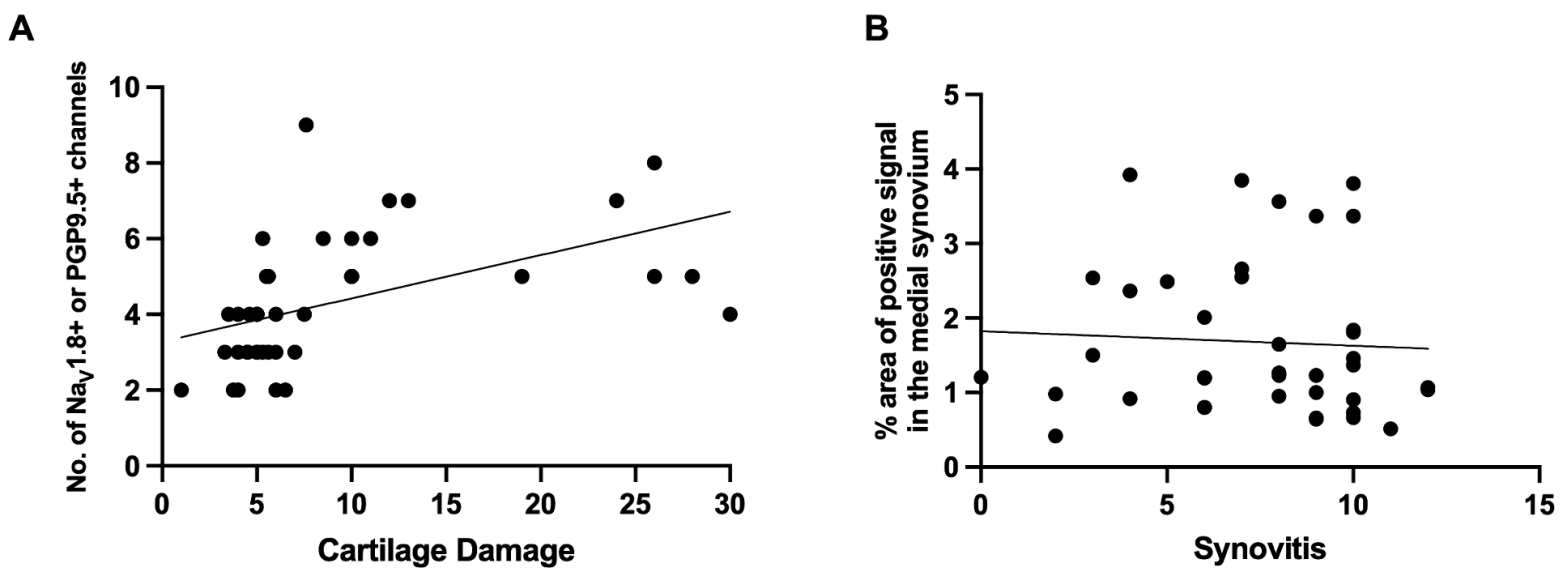
**(A)** Correlation between Na_V_1.8+ or PGP9.5+ medial subchondral bone channels and cartilage degeneration [*r* = 0.664, 95% confidence interval 0.434 to 0.813, P (two-tailed) <0.0001, Number of XY Pairs 39]. **(B)** Correlation between Na_V_1.8+ or PGP9.5+ signal in the medial synovium to synovitis scores in the medial synovium [*r* = −0.117, 95% confidence interval − 0.425 to 0.216, P (two-tailed) = 0.4787, Number of XY Pairs 39].

In summary, the subchondral bone channels containing nociceptors appeared early and progressed with worsening cartilage damage. Correlation analysis revealed strong correlation between subchondral bone nerves and cartilage damage (*r* = 0.664, 95% confidence interval 0.434 to 0.813, P (two-tailed) <0.0001, Number of XY Pairs 39) (Figure 5A). In contrast, no correlation was found between synovial nociceptor sprouting and synovitis (*r* = −0.117, 95% confidence interval −0.425 to 0.216, P (two-tailed) = 0.4787, Number of XY Pairs 39) (Figure 5B), reflecting the fact that synovial sprouting peaks early on and does not change as the diseases progresses, whereas synovitis peaks early but subsides at later stages of the disease.

## Discussion

Knee joints are abundantly innervated by nociceptors, but detailed information on the distribution of free nerve endings in the knee joint is scarce, contributing to our incomplete understanding of what drives joint pain. Motivated by increasing evidence that OA of the knee is accompanied by structural plasticity of intra-articular nociceptors, in human subjects as well as in experimental models (Loeser et al., 2012; Aso et al., 2020, 2022; Ishihara et al., 2021), we performed a qualitative and quantitative analysis of the joint nociceptive innervation in four mouse models of knee OA, so as to identify common aspects of neuroplasticity that accompany OA. We documented the anatomical distribution of knee joint nociceptors in early and late-stage joint disease in two surgical models, a non-invasive model of PTOA, and in primary age-associated OA.

The key finding in this study is that nociceptor sprouting in the synovium and in subchondral bone channels occurred across all 4 models, in a manner that is tightly linked to joint pathology. In both surgical models and in the non-invasive PTOA model, OA joint damage was restricted to the medial compartment, as was nociceptor sprouting. In contrast, primary age-associated OA affected both compartments, with the medial compartment more severely affected. This was mirrored by nociceptor sprouting, which occurred in both compartments but was more pronounced in the medial compartment.

In the synovial membrane, neo-innervation occurred in a temporal manner matching the time-course of synovial pathology. Nociceptor sprouting had plateaued by the earlier timepoint (week 4 in the surgical models and week 1 after ACLR), consistent with peak synovitis in the early stages of these models. In all three induced models, synovitis decreased by the later timepoints, while nociceptor density remained unchanged. Synovitis scores have been previously reported to peak early on in these models, including 4 weeks after DMM (Shu et al., 2020), and in two models of ACL injury, surgically anterior cruciate ligament transection and ACLR (Urban et al., 2020). Finally, old mice showed mild synovial changes, confirming our previous findings (Geraghty et al., 2023), and this was paralleled by neuronal sprouting in the synovium. Conflicting findings have been reported in the synovium, however, with either increased nociceptive innervation in surgical models (Obeidat et al., 2019; Aso et al., 2022), a transient or permanent reduction of innervation in collagenase induced OA (Buma et al., 1992; Murakami et al., 2015), or no changes in mechanically loaded murine joints (Ter Heegde et al., 2020). In human OA synovium, a reduction in PGP9.5 and CGRP-fibers has been described, close to the lining layer (Eitner et al., 2013). This may be attributable to varying inflammatory components in different models, as well as lack of data covering different stages of joint damage and different anatomical locations in the knee joint. Indeed, most published studies analyzed joints with end-stage OA, including in human knees, which are often collected at time of total joint replacement (Eitner et al., 2013; Aso et al., 2019, 2020).

Neuronal sprouting within subchondral bone channels also started early on, but in contrast to synovial sprouting, it progressed with worsening joint damage. We detected neurons in subchondral bone channels, and these positive channels were significantly greater in number, longer, and closer to the tidemark at later timepoints compared to earlier timepoints and age-matched controls. Changes in subchondral bone innervation were previously reported in a rat medial meniscectomy model, where CGRP-fibers within osteochondral channels were detected as early as 2 weeks after surgery, and increased in density at later stages (Aso et al., 2022). Subchondral bone neuroplasticity was first reported in human OA knees, where CGRP fibers were detected within osteochondral channels, and—as in the rat—were associated with pain (Aso et al., 2020). Interestingly, we found that severe osteoarthritic changes in late-stage OA were associated with more branching of sprouted nerves within channels, which may result in an increased receptive field, as has been described in the skin (Peng et al., 1999).

Importantly, all 4 models showed abundant nociceptors within bone marrow cavities in osteophytes. Large bundles of multiple sensory nerve fibers have been described within bone marrow cavities in several post-mortem axial and appendicular bones (Steverink et al., 2021). Nociceptor sprouting was only observed in relatively mature, mineralized osteophytes, and not in precursor chondrophytes. This indicates that mineralization, angiogenesis, and nerve sprouting are potentially coupled processes in osteophytes. PGP9.5 nerve trunks have also been described in subchondral bone marrow and within osteophyte marrow cavities in knees after joint replacement surgery for tibiofemoral OA (Suri et al., 2007).

In summary, our findings suggest that experimental OA is accompanied by profound neuronal remodeling in different joint tissues, starting early after OA induction and associated with disease progression. These observations, together with reports in human knees, suggest that nociceptor sprouting is a consistent anatomical hallmark of OA, intrinsic to joint pathology. It also appears that neuronal sprouting starts early in the disease, both in the synovium and in the subchondral bone, and is established more rapidly in the synovium than in the bone. This is consistent with a recent study in rats, which reported that CGRP+ synovial innervation was increased at early stages (2 weeks after medial meniscectomy) and then decreased at later stages, while the sprouting in the subchondral bone continued to increase over time (Aso et al., 2022). In older mice, where we have previously reported that the existing nociceptor innervation in the lateral synovium declines between the ages of 10 and 26 weeks (Obeidat et al., 2019), we observed that by age 2 years, sprouting accompanied mild OA joint damage.

These observations raise the question as to how this neuronal plasticity might relate to pain. In all four models, our group and others have shown that these mice develop pain-related behaviors with progressive disease (Knights et al., 2012; Miller and Malfait, 2017; von Loga et al., 2020; Bergman et al., 2023; Geraghty et al., 2023). Since we have not yet published our findings in the male PMX model, we included a detailed time-course testing for knee hyperalgesia and weight bearing (Supplementary Figure S10). We found that the mice developed knee hyperalgesia 4 weeks after PMX but not sham surgery, and weight bearing asymmetry 10 and 12 weeks after PMX. It would be reasonable to conclude that Na_V_1.8+ neo-innervation contributes to pain, as is suggested by the fact that the intra-articular administration of lidocaine reverses knee hyperalgesia in the DMM model (Miller et al., 2018). Definite proof of this will require sophisticated approaches to selectively ablate these particular neurons, and assess the effect on pain. Our findings might be discussed in the context of a recent study that utilized *in vivo* electrophysiology to document responses of knee-innervating and bone-innervating neurons in the rat monosodium iodoacetate (MIA) model, which compared responses at an early (day 3) and a late timepoint (day 28) (Morgan et al., 2022). This study reported that early pain involved activation and sensitization of nerves within the medial aspect of the joint capsule, which is lined with the synovium where we detected substantial neuronal sprouting at early stages of pathology across all models. Moreover, the authors reported that pain in late-stage MIA was associated with recruitment of nerves in the subchondral bone (Morgan et al., 2022). In addition, a study in knees of patients undergoing total knee replacement for painful knee OA reported increased CGRP+ osteochondral channels compared to asymptomatic *post-*mortem controls, suggesting that these nociceptors in the subchondral bone contribute to pain in late-stage OA (Aso et al., 2020).

Anatomical neuroplasticity in the OA joint may open new avenues for targeting joint pain and it will be critical to identify the factors that drive neuroplasticity and neoinnervation. One potential candidate to explain nerve sprouting in OA models is the neurotrophin, nerve growth factor (NGF), which has been shown to promote nerve sprouting in bone cancer and inflammatory models (Jimenez-Andrade et al., 2010; Ghilardi et al., 2012). NGF is expressed in the synovium and in osteochondral channels in OA joints (Mapp et al., 2008; Stoppiello et al., 2014; Aso et al., 2022). Clinical trials with tanezumab, a monoclonal antibody that neutralizes NGF, showed promising results in treating OA pain but the occurrence of rapidly progressive OA (RPOA) in some patients halted the development of these antibodies (Wise et al., 2021). The occurrence of RPOA with anti-NGF treatment raises the possibility that the sprouting nociceptors might also be important for joint homeostasis and maintenance of joint health, but this has yet to be proven. Future studies will explore the functional significance of newly sprouted nerves by selective ablation of knee nociceptors and study the effect on joint integrity and OA pain.

A limitation of our study is the fact that all experiments were only conducted in male mice. We elected to focus on male mice, since our original studies were performed in the DMM model, where females develop barely any joint damage (Ma et al., 2007). Furthermore, male mice are far more susceptible to primary age-associated OA then females (Walton, 1977; Geraghty et al., 2023). Ongoing studies in the PMX and in the ACLR model suggest that sprouting also occurs in female mice (Obeidat, 2021; Bergman et al., 2023), and future studies will investigate sex-specific processes. Aging studies were performed in WT mice stained for the pan-neuronal marker, PGP9.5, due to the unavailability of 2-year-old Na_V_1.8 reporter mice when we performed these studies. Detailed studies on aging Na_V_1.8 tdTomato reporter mice of both sexes are currently ongoing.

The tight relationship between joint pathology and neuronal sprouting suggests that neuroplasticity is a fundamental component of OA joint pathology. Together with the reports of neuronal plasticity in human knee joints, our findings warrant an in-depth exploration of the innervation of the OA knee joint, the drivers of the observed neuroplasticity, and the communication between joint nociceptors and the joint tissues they innervate. Understanding the exact nature of this plasticity and how it comes about, is critical if we are to make progress in the treatment of pain in osteoarthritis.

## Data availability statement

The original contributions presented in the study are included in the article/Supplementary material, further inquiries can be directed to the corresponding author.

## Ethics statement

The animal study was approved by Institutional Animal Care and Use Committee at Rush University Medical Center or the University of Michigan. The study was conducted in accordance with the local legislation and institutional requirements.

## Author contributions

AO: Conceptualization, Data curation, Formal analysis, Investigation, Methodology, Project administration, Resources, Software, Supervision, Validation, Visualization, Writing – original draft, Writing – review & editing, Funding acquisition. SI: Data curation, Methodology, Writing – review & editing. JL: Data curation, Methodology, Writing – review & editing. NA: Data curation, Methodology, Writing – review & editing. LL: Data curation, Methodology, Writing – review & editing. LJ: Data curation, Writing – review & editing. TM: Funding acquisition, Writing – review & editing. RJM: Conceptualization, Funding acquisition, Resources, Writing – review & editing. REM: Conceptualization, Data curation, Formal analysis, Funding acquisition, Investigation, Methodology, Project administration, Resources, Software, Supervision, Validation, Visualization, Writing – original draft, Writing – review & editing. A-MM: Conceptualization, Data curation, Formal analysis, Funding acquisition, Investigation, Methodology, Project administration, Resources, Software, Supervision, Validation, Visualization, Writing – original draft, Writing – review & editing.
