# Supplementary material for: Intra-articular sprouting of nociceptors accompanies progressive osteoarthritis: comparative evidence in four murine models
Source: Front Neuroanat. 2024 Jul 15;18:1429124. doi: 10.3389/fnana.2024.1429124 (PMC11284167; doi:10.3389/fnana.2024.1429124)
Supplement: Supplementary file 1 [file Data_Sheet_1.PDF]

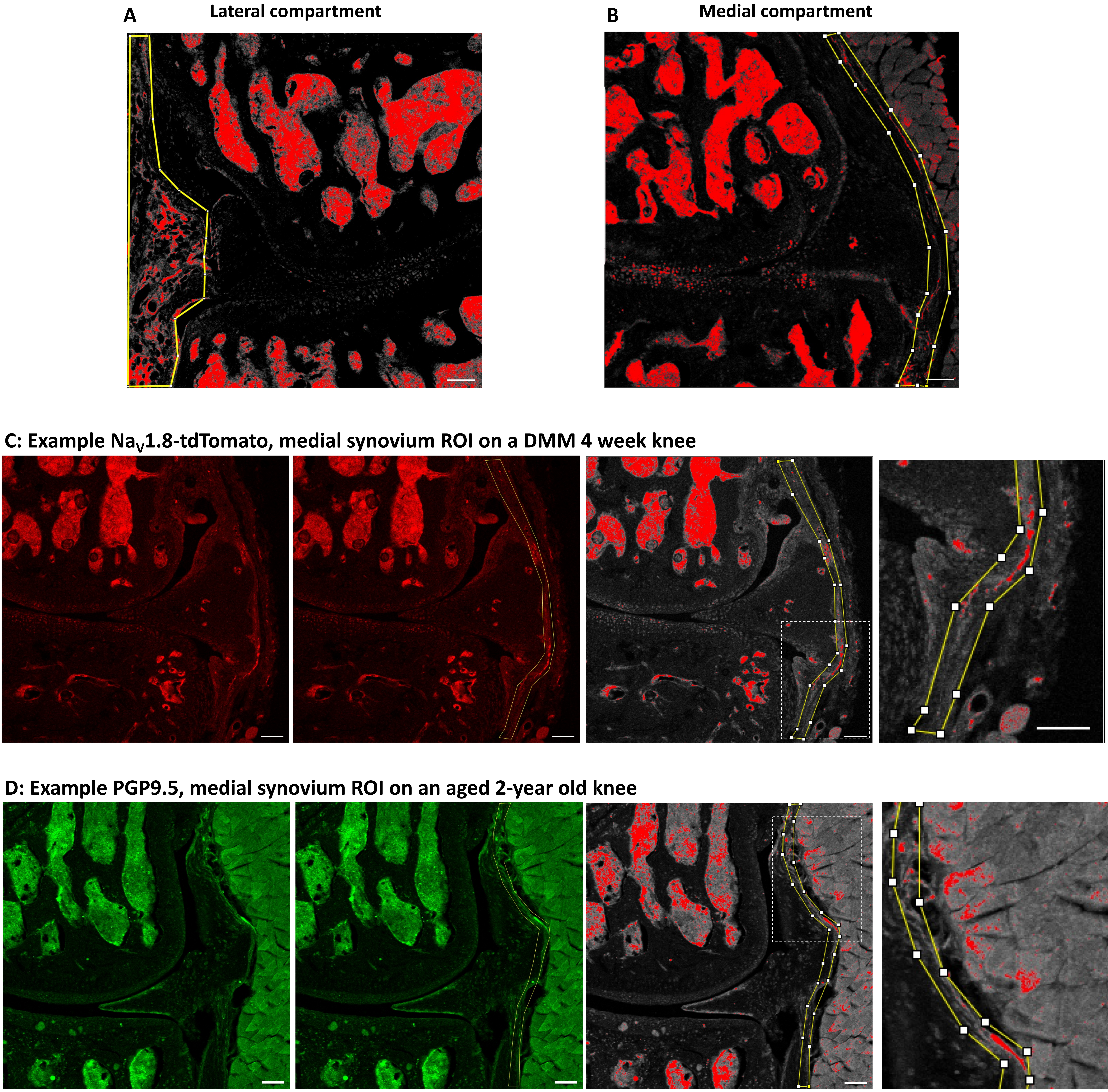

Suppl. Figure 1: Outline of regions of interest for quantification of signal in mid-joint coronal sections. Thresholds were adjusted for all images similarly to control for background. The area of positive signal within each ROI was measured and was normalized to the total area of the ROI; the percentage of positive signal per ROI is reported.. (A) The lateral synovium outlined in a knee 4-weeks after DMM surgery; (B) The medial synovium adjacent to the capsule outlined in a knee in a knee 8-weeks after DMM surgery. ROI in yellow, positive signal within the ROI in black. Scale bar = 100 µm. (C) Example Na_V1.8-tdTomato knee from a mouse 4 weeks after DMM surgery. From left to right: Image adjusted as images shown in Fig 2; Image+ROI; Thresholded image and area of positive signal within the ROI calculated as above; inset of the thresholded image to illustrate the morphology of structures with positive signal after thresholding. (D) Example PGP9.5 stained images from a 2-year old mouse knee, images left to right as in (C).

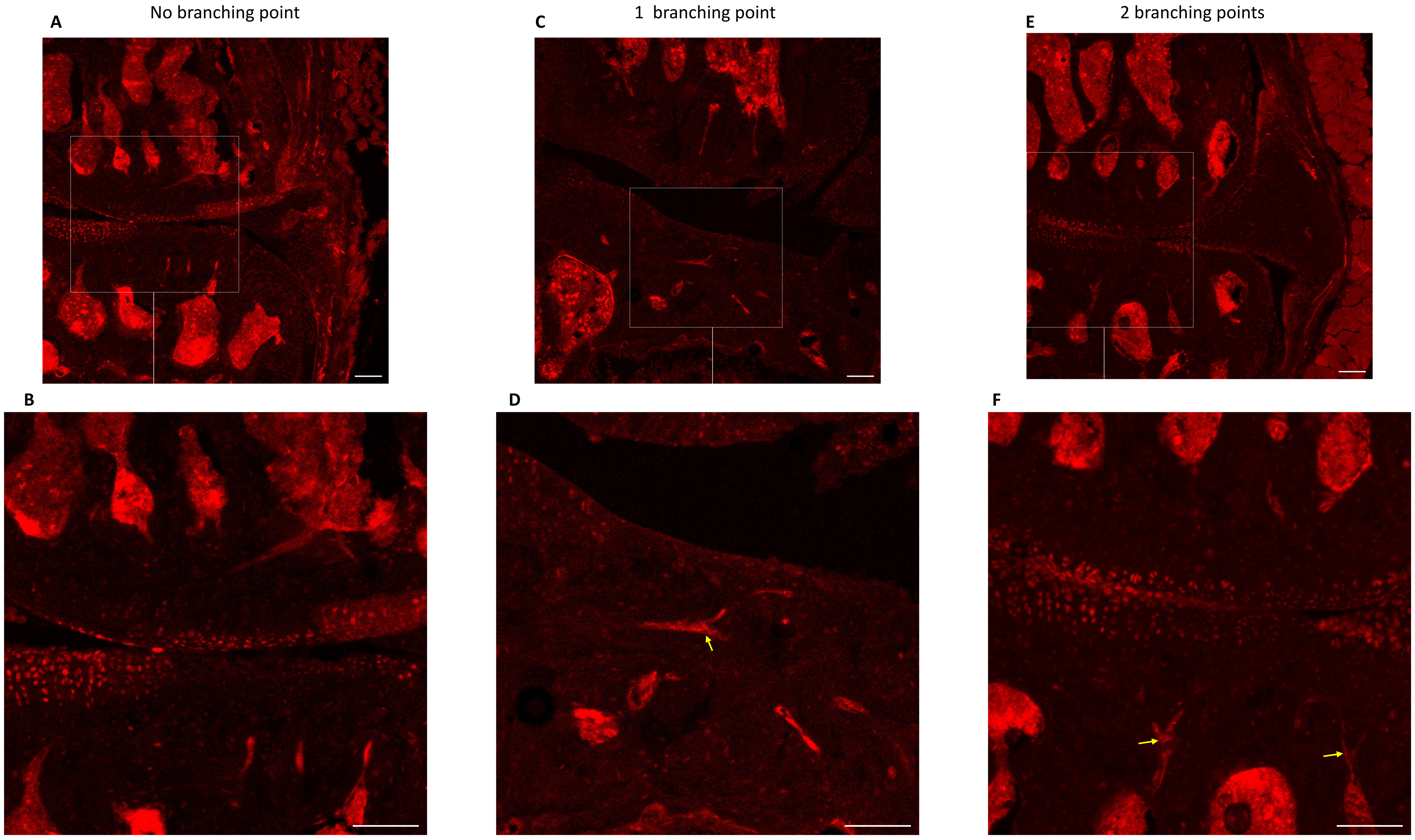

Suppl. Figure 2: Quantification of branching points in $Na_V1.8+$ subchondral bone channels. A and B (zoomed in) show a confocal images with no branching points; C and D show an image with one branching point, indicated by the yellow arrow; E and F show a confocal image with two branching points, indicated by the yellow arrows. Scale bar =100 µm.

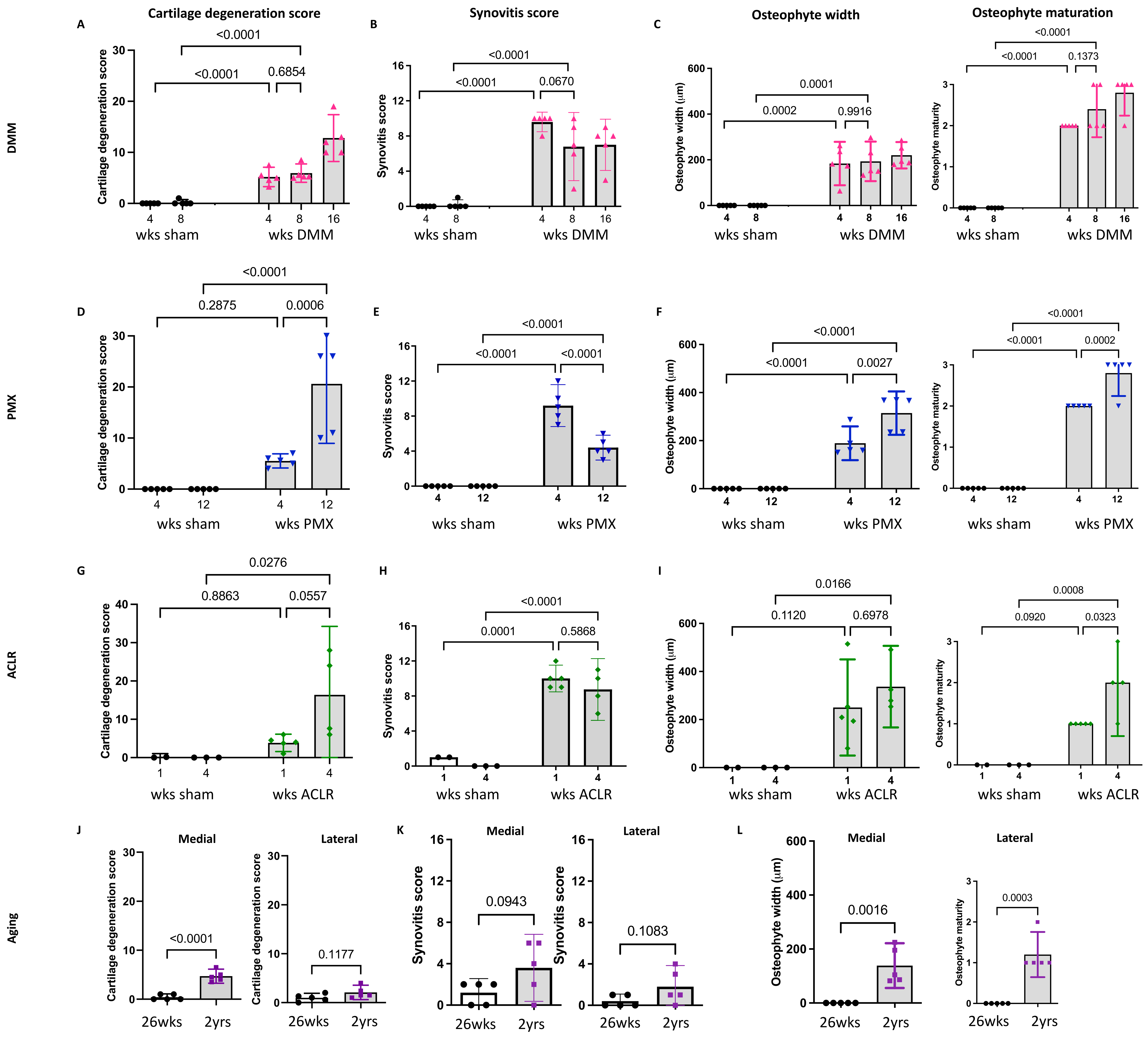

Suppl. Figure 3: (A-C) Medial cartilage degeneration scores, synovitis scores, osteophyte width (measured in µm), and osteophyte maturity in right knees of 4, 8 and 16 weeks after sham or DMM surgery in male mice; (D-F) Scores in right knees 4 and 12 weeks after sham or PMX in male mice; (G-I) Scores in right knees 1 and 4 weeks after sham or ACLR in male mice; (J-L) Scores in right knees of 26-week old and 2-year old naïve male mice. Wks=weeks. Medial cartilage degeneration DMM 4wks = 5.18±1.5, sham 4wks = 0; DMM 8wks = 6±1.4, sham 8wks = 0.2±0.4; DMM 16+wks = 12.8±3.7; PMX 4wks = 5.6±1.2, sham 4wks = 0, PMX 12wks = 20.6±9.3, sham 12wks = 0; ACLR 1wk = 3.85±1.8, sham 1wk = 0.07±0.1; ACLR 4wks = 16.4±11.2, sham 4wks = 0. Medial cartilage degeneration score for 26-wk old mice = 0.4±0.55, and lateral cartilage degeneration score for 26-wk old mice = 0.98±0.74; Medial cartilage degeneration score for 2-year old mice = 4.72±1.19, and lateral = 2.08±1.18. Synovitis scores are sum scores of synovial hyperplasia, cellularity, and fibrosis for the medial compartment of the right knees. Osteophyte width was measured in µm for the major osteophyte in the medial compartment. Osteophyte width for DMM 4 weeks= 183.9±76.6, DMM 8 weeks= 193.2±69.6, PMX 4 weeks= 189.2±56.6, PMX 12 weeks= 314.7±72.7, ACLR 1 week= 250.2±161.3, ACLR 4 weeks= 336.9.±106.5, 2-year old naïve mice= 138.8±66.8. All graphs show the mean ± 95% CI.

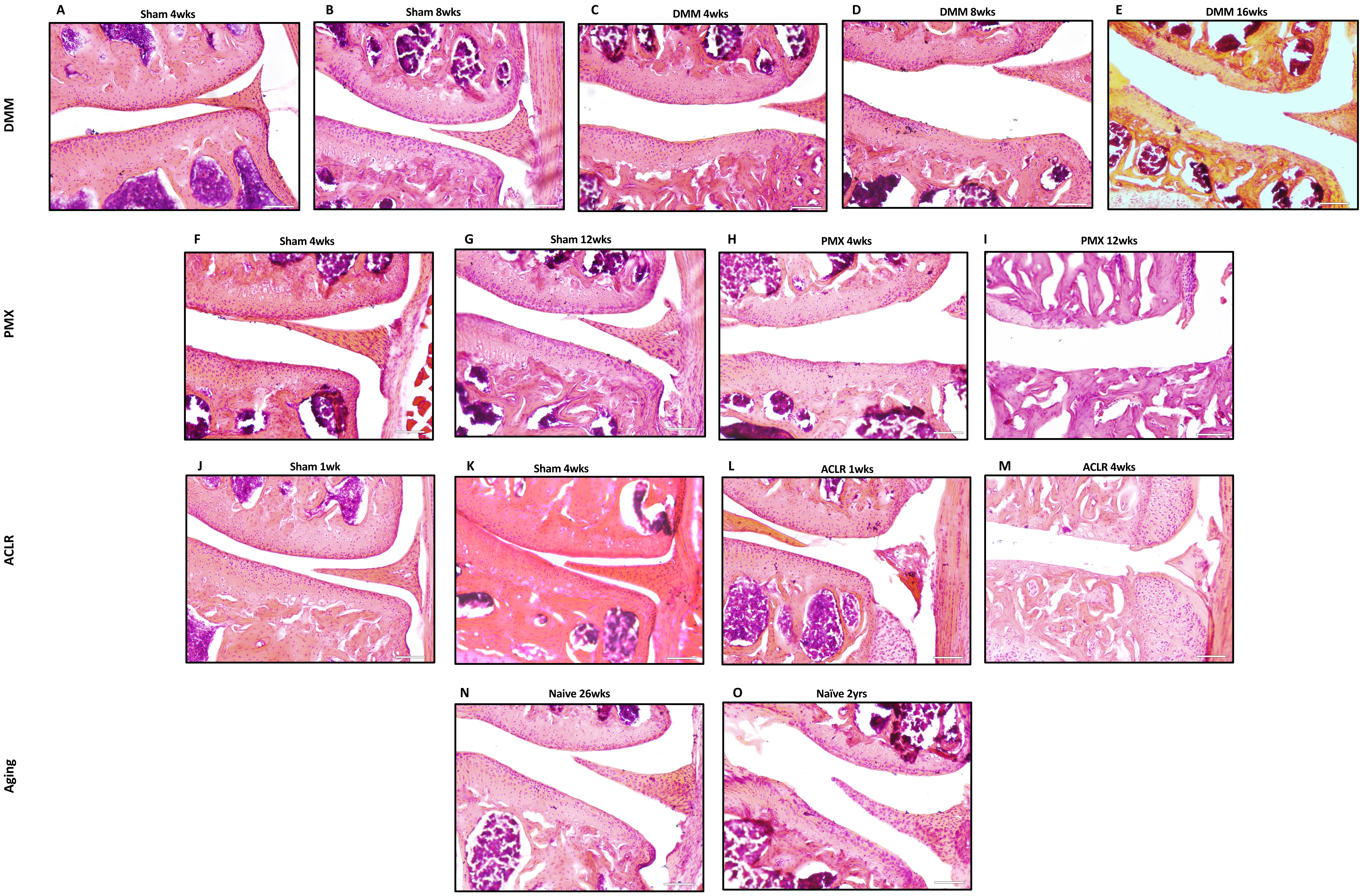

Suppl. Figure 4: Representative histological images of the medial compartment of Na_V1.8-tdTomato and WT knees (A-E) at 4 and 8 after sham and 4, 8 and 16 after DMM surgery; (F-I) 4 and 12 weeks after sham or PMX surgery; (J-M) 1 and 4 weeks after sham or ACLR; (N,O) knees from 26 week-old and 2 year-old naïve mice. Scale bar = 100 µm.

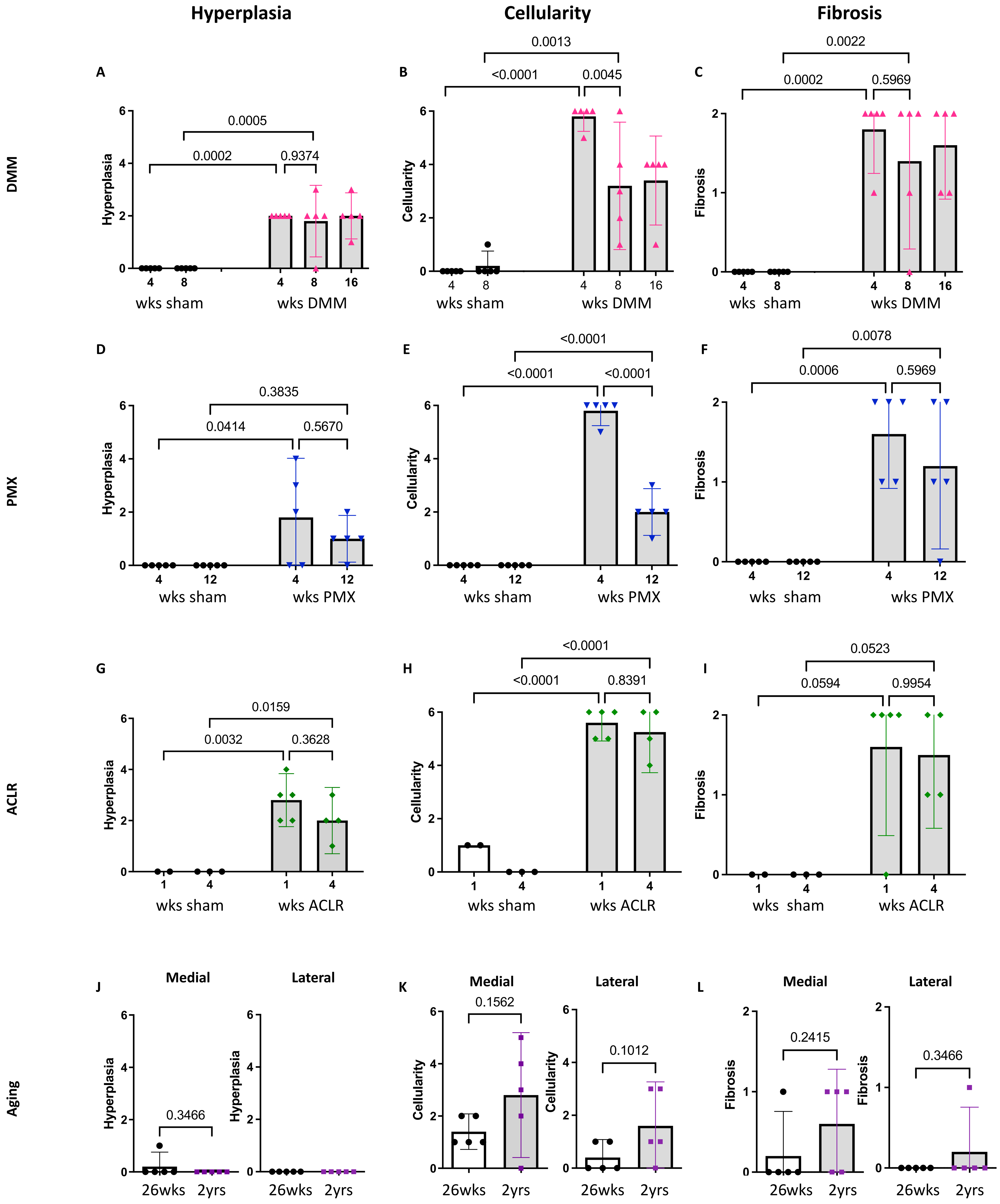

Suppl. Figure 5: Synovial scoring of hyperplasia, cellularity and fibrosis, respectively, including total scores from medial femoral and medial tibial joint spaces. (A-C) 4, 8 and 16 weeks after sham or DMM surgery; (D-F) 4 and 12 weeks after sham or PMX surgery; (G-I) 1 and 4 weeks after sham or ACLR injury; (J-L) 26-week old and 2-year old naïve mice. Mean ± 95% CI.

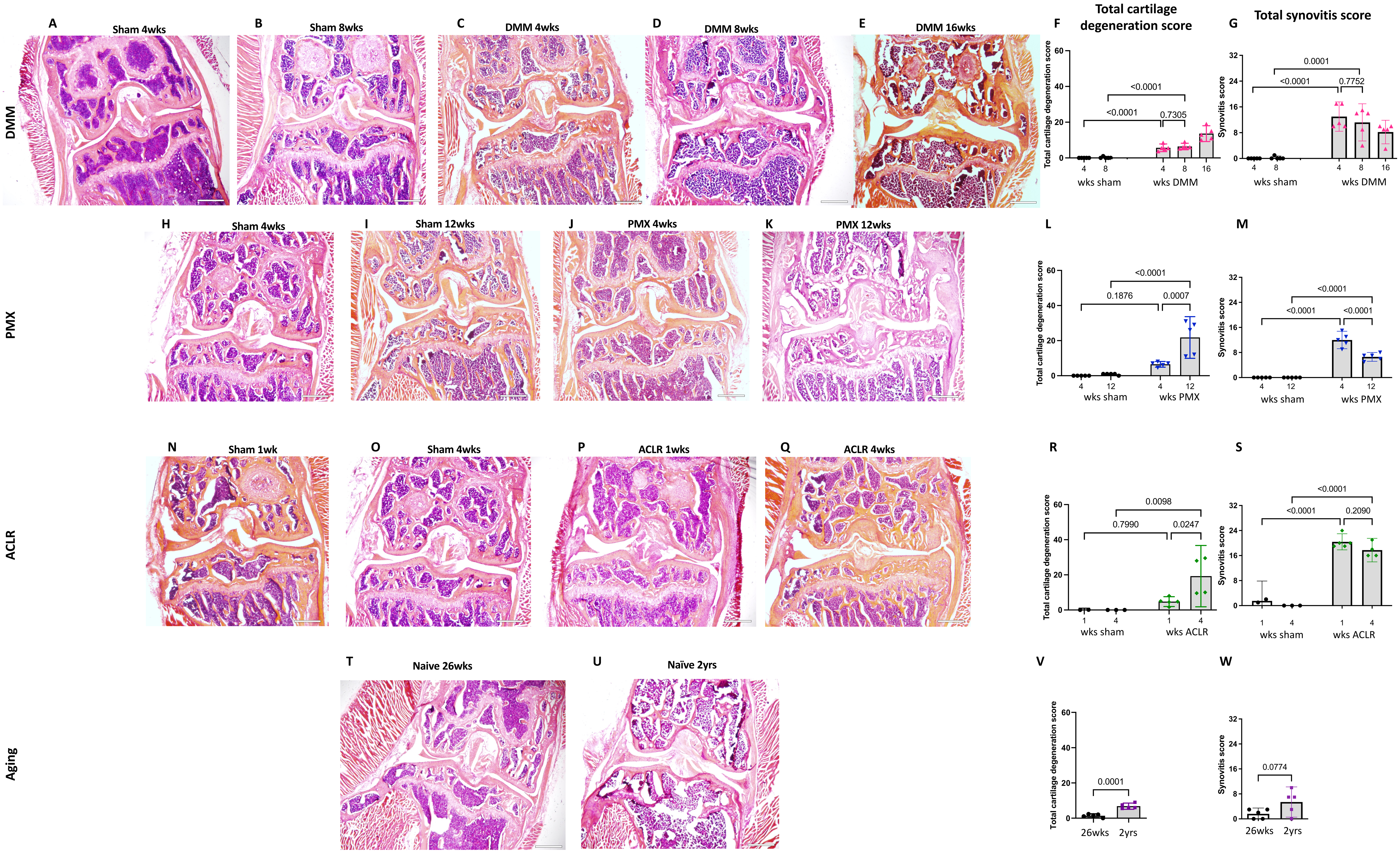

Suppl. Figure 6: Representative histological images of whole knees of Na_V1.8-tdTomato and WT mice (A-E) 4, 8, and 16 weeks after sham or DMM surgery; (F,G) total cartilage degeneration score and synovitis total limb score, respectively; (H-K) 4 and 12 weeks after sham or PMX surgery; (L,M) total cartilage degeneration score and synovitis total limb score, respectively; (N-Q) 1 and 4 weeks after sham or ACLR injury; (R,S) total cartilage degeneration score and synovitis total limb score, respectively; (T-U) knees from 26-week old and 2-year old naïve mice; (V,W) total cartilage degeneration score and synovitis total limb score, respectively. Mean ± 95% CI. Scale bar = 500 µm.

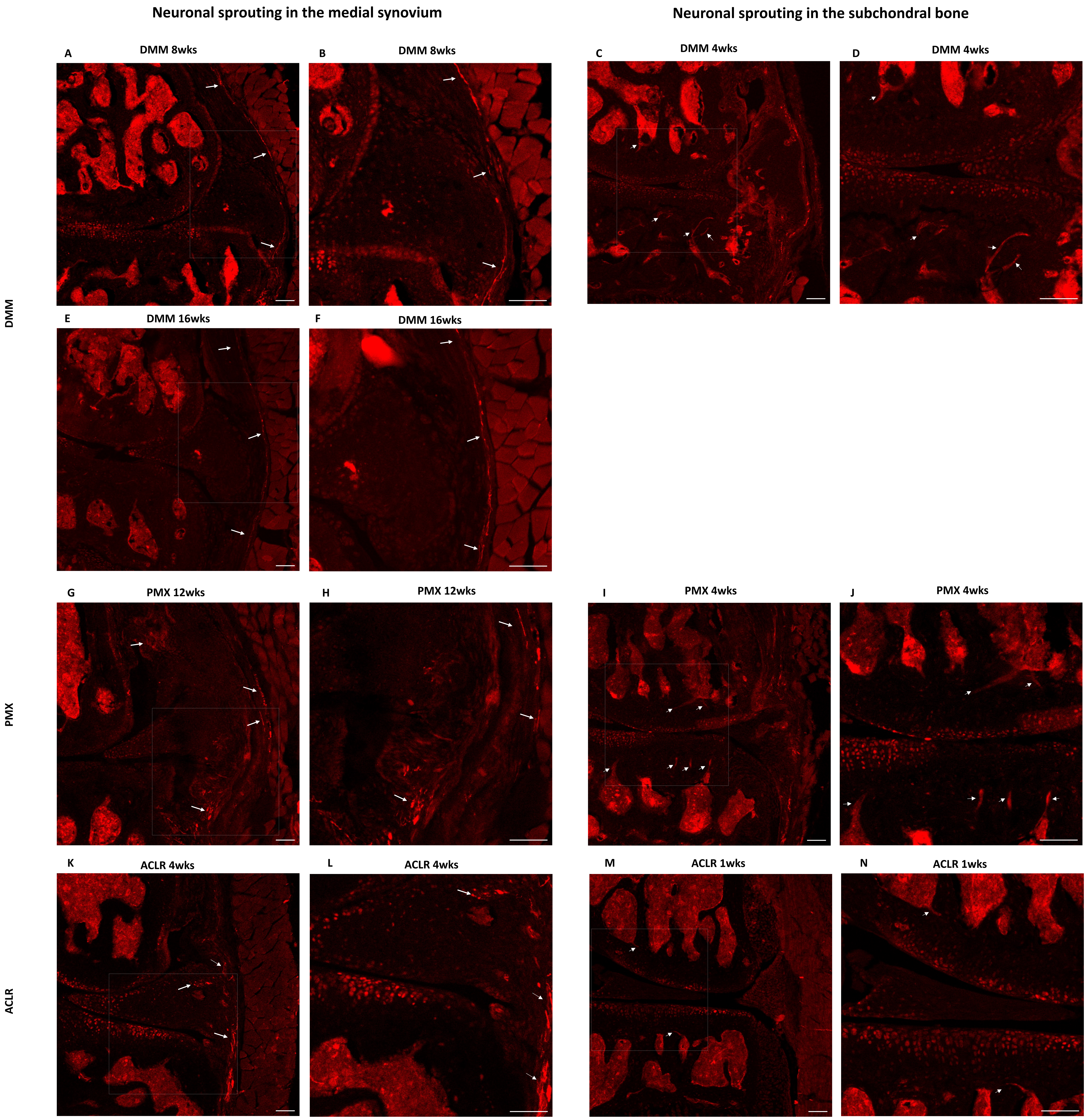

Suppl. Figure 7: Representative confocal images of $Na_V1.8$-tdTomato mouse knees showing (A,B,E,F) neoinnervation in the medial synovium (white arrows) 8 and 16 weeks after sham and DMM surgery; (C,D) nerve fibers in subchondral bone channels (white arrows) 4 weeks after sham and DMM surgery; (G,H) nerve fibers in the medial synovium 12 weeks after sham and PMX surgery; (I,J) nerve fibers within subchondral bone channels 4 weeks after sham and PMX surgery; (E,L) nerve fibers in the medial synovium 4 weeks after sham and ACLR injury; (M,N) nerve fibers within subchondral bone channels 1 week after sham and ACLR injury. Scale bar = 100 µm.

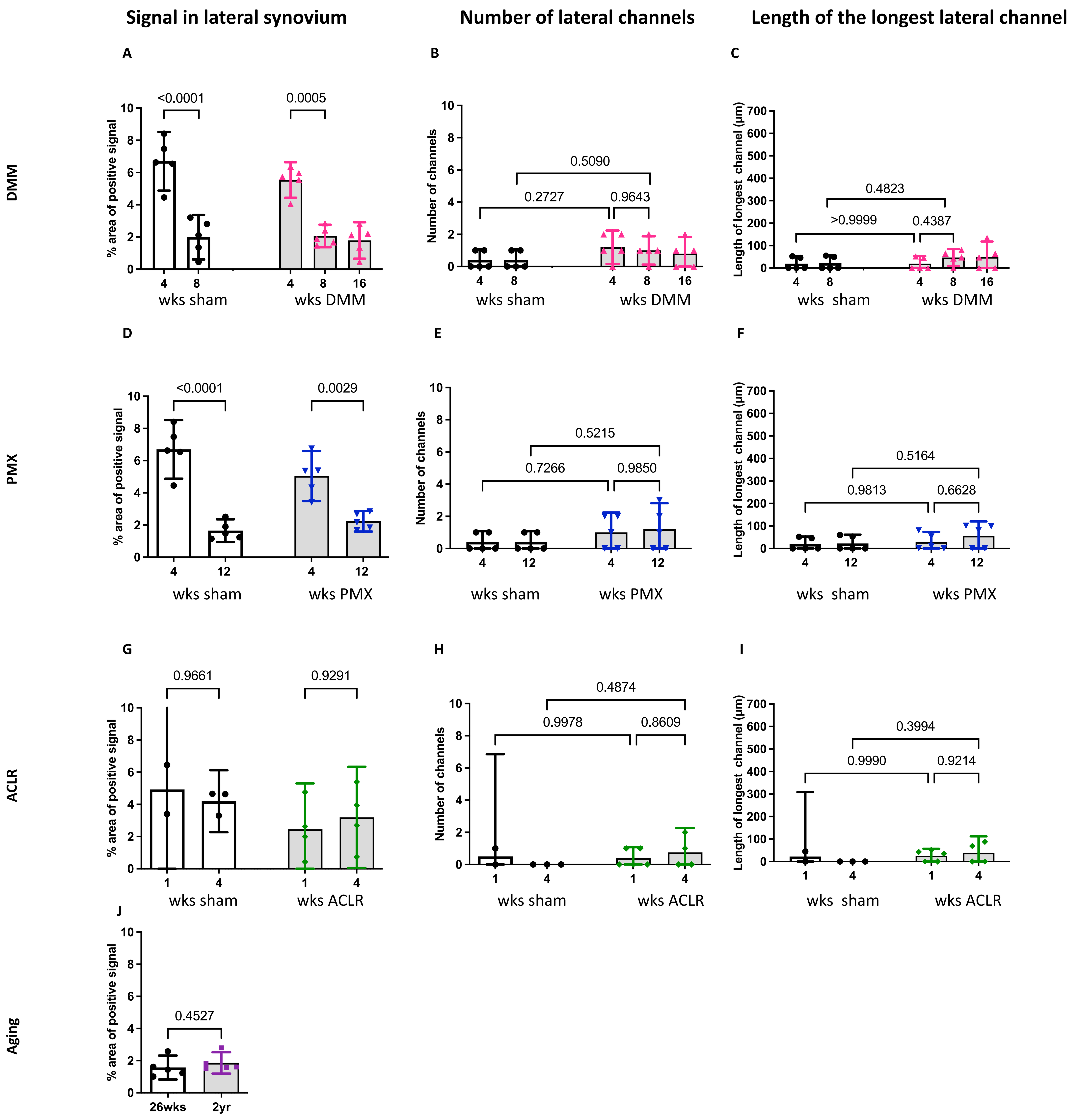

Suppl. Figure 8: Quantification of Na$_V$1.8+ signal in the lateral synovium, the number of lateral subchondral bone channels and the length of the longest channel at (A-C) 4, 8 and 16 weeks after DMM or sham surgery; (D-F) 4 and 12 weeks after PMX or sham surgery; (G-I) 1 and 4 weeks after ACLR injury or sham; (J) 26-week old and 2-year old naïve mice. Mean ± 95% CI.

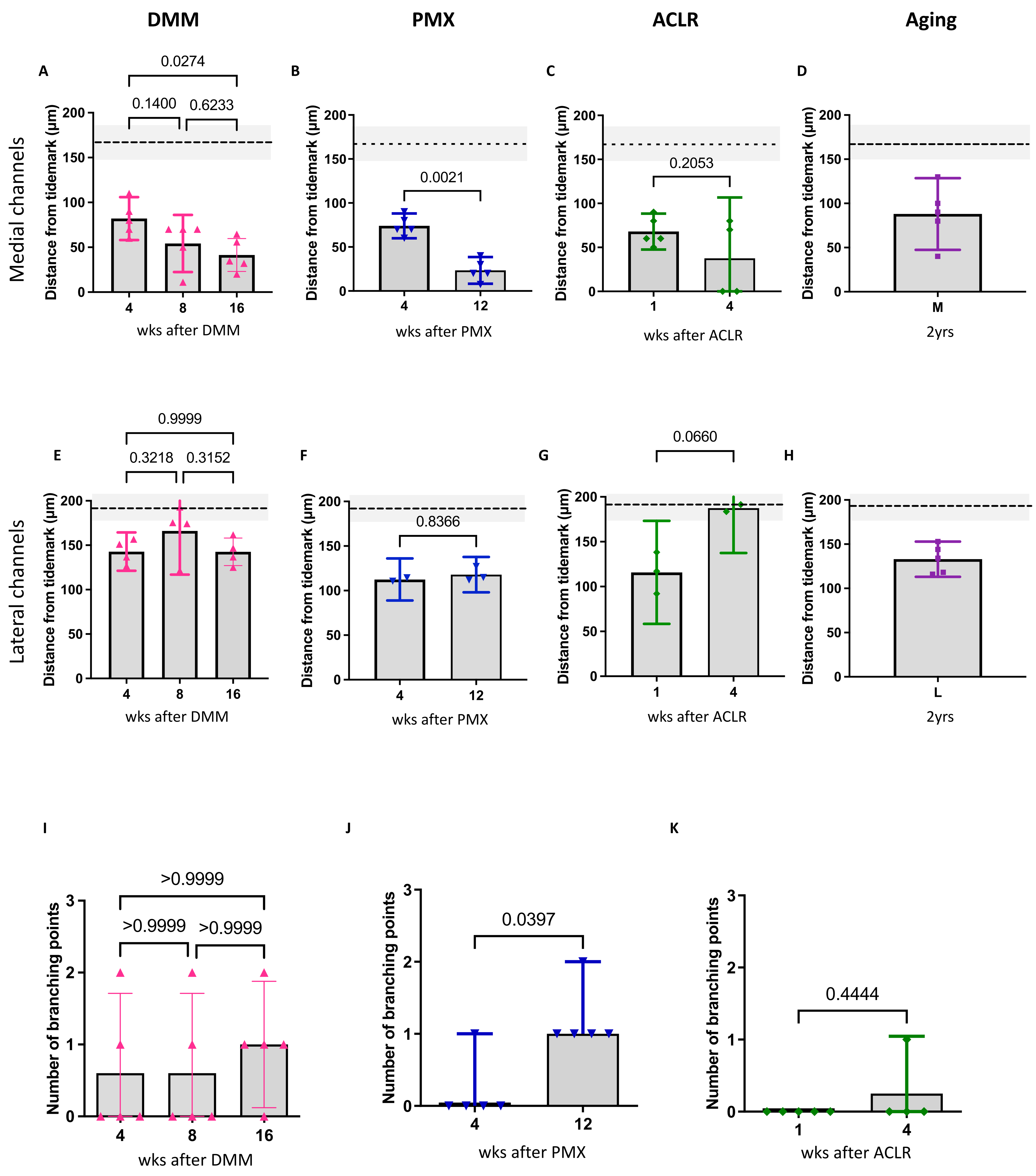

Suppl. Figure 9 : (A-D) Quantification of the distance of Na_V1.8+ and PGP9.5+ medial channels from the tidemark; (E-H) Quantification of the distance of Na_V1.8+ and PGP9.5+ lateral channels from the tidemark; (I-K) Number of branching points 4, 8 and 16 weeks after DMM, 4 and 12 weeks after PMX surgery, and 1 and 4 weeks after ACLR injury. Dotted line = average of distance from tidemark for naïve and sham mice; shading = standard deviation. M=medial, L=lateral; (A-H) Mean ± 95% CI. (I-K) Median ± 95% CI.

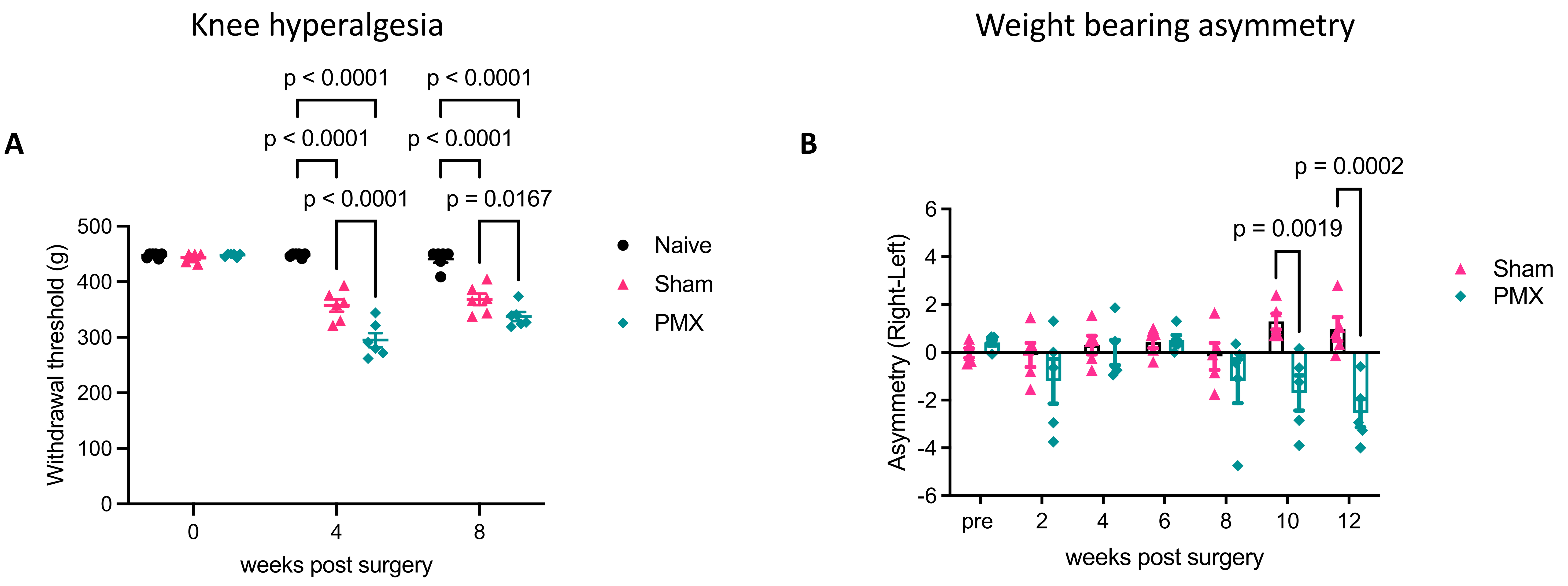

Suppl. Figure 10: (A) Knee hyperalgesia for male wildtype mice at 0, 4 and 8 weeks after PMX or sham surgery and age matched naïve mice (n=6); Two-way ANOVA with Tukey post-hoc test. (B) Weight-bearing asymmetry for mice between 0 to 12 weeks after PMX or sham surgery (n=5); Two-way ANOVA with Sidac post-hoc test.
