# Supplementary material for: Intra-articular sprouting of nociceptors accompanies progressive osteoarthritis: comparative evidence in four murine models
Source: Front Neuroanat. 2024 Jul 15;18:1429124. doi: 10.3389/fnana.2024.1429124 (PMC11284167; doi:10.3389/fnana.2024.1429124)
Supplement: Supplementary file 2 [file Data_Sheet_2.docx]

**Supplementary Methods**

**Animals** –Na_V_1.8Cre-tdTomato reporter mice (a gift from Dr. John Wood, University College London, London, UK) express a bright red fluorescent tdTomato reporter in all neurons that express the voltage-gated sodium channel, Na_V_1.8. This neuronal subset comprises approximately 75% of dorsal root ganglion (DRG) sensory neurons, including >90% of C-fibers, as well as a fraction of Aδ-nociceptors (1,2).

**Destabilization of the medial meniscus (DMM)** – Mice were anesthetized by inhalation of isoflurane. The joint capsule was opened and the anterior medial meniscotibial ligament was severed. The knee was flushed with saline, and the incision closed. Sham surgery was identical to DMM, except that the ligament was left intact (3).

**Partial meniscectomy (PMX)** – Mice were anesthetized by inhalation of isoflurane. After medial parapatellar arthrotomy, the infra-patellar fat pad was dissected to expose the anterior part of the medial compartment of the knee. The medial meniscotibial ligament was transected to release the anterior horn of the medial meniscus, and approximately 1/3 to 1/2 of the anterior portion of medial meniscus was cut. For sham surgery, the medial meniscotibial ligament and the medial meniscus were left intact (4).

**Anterior cruciate ligament rupture (ACLR)** – ACLR mice were subjected to a tibial compression-based noninvasive ACL rupture protocol, as described (5). Briefly, mice were anesthetized with 2% inhaled isoflurane and immobilized on a custom fixture on a mechanical testing system (Electroforce 3300AT, TA Instruments, New Castle, DE). The right knee was flexed to 100^o^ and secured within a trough to restrict medial-lateral motion. The hindpaw was secured in 30^o^ dorsiflexion. After preloading and conditioning, a rapid displacement of 1.5 mm (10 mm/s) was applied via the hindpaw, causing tibial compression, anterior tibial subluxation, and ACL rupture. Complete ACL rupture was confirmed via an anterior drawer test. Following the rupture or sham procedure, animals were administered a single dose of subcutaneous carprofen (5 mg/kg). Mice were allowed *ad libitum* cage activity and provided unlimited access to food and water. Mice were housed in ventilated cages containing a maximum of 5 animals (randomized mix of Sham and ACLR) and maintained within a 12- hour light/dark cycle facility (6). Sham and ACLR mice were randomized using computer-aided block randomization. within the ACLR group, mice were further randomized to two study timepoints, 1 week (n=5) or 4 weeks (n=4).

**Knee histology and immunohistochemistry** – Mice were transcardially perfused, and right knees were collected. Knees were decalcified in 14% EDTA for 2-3 weeks and cryopreserved using 30% sucrose. Twenty µm-thick coronal sections were then collected from the mid-joint area as previously described (7). Knee sections from WT mice were stained for the pan-neuronal marker, protein gene product 9.5 (PGP9.5), as described (7). Sections were blocked and incubated overnight with a primary antibody against PGP9.5 (rabbit polyclonal antibody, Sigma-Aldrich, SAB4503057; 1:100) at 4 °C, followed by a secondary antibody, anti-rabbit conjugated Alexa Fluor 633 (Molecular Probes, 1:500). Control sections were treated the same but without primary antibody.

**Knee hyperalgesia** – Knee hyperalgesia was measured using Pressure Application Measurement (PAM) device as previously described (8,9). Briefly, after restraining animals, the PAM transducer was pressed against the medial side of flexed ipsilateral knee.  PAM software guided the user to apply a constantly increasing force (30 g/s) up to a maximum of 450 g. The force is recorded when the mouse tries to withdraw the knee. Two measurements were obtained per knee, and the withdrawal threshold were averaged and reported. Knee hyperalgesia was measured at baseline before surgery and at weeks 4 and 8 after PMX or sham surgery and age matched naïve mice (n=6/group). Knee hyperalgesia was assessed by a blinded observer (SI).

**Weight bearing asymmetry** – Weight bearing asymmetry was assessed utilizing a custom voluntarily accessed static incapacitance (VASIC) method where mice were trained to perform a string-pulling task (10) while freely standing on a static incapacitance meter platform. To accustom mice to the task, a training phase and a testing phase were necessary. The night before each phase, food was removed to increase motivation to the task. Mice were trained to the task in a cage void of stimuli other than 20 strings of varying lengths (53–75 cm) hanging from the wire cage top. Ten of the strings were baited with half of a Honey Nut Cheerio™ to serve as a reward. During the training phase, mice were given one hour to pull all 20 strings, and mice unable to pull at least 15 were retrained. The testing phase occurred the following week in which mice were placed in a custom-built plexiglass chamber atop a Bioseb Static Incapacitance meter (Harvard Apparatus). Weight bearing was recorded once mice had both hind limbs placed on each load cell and were pulling a hanging string (100 cm) to receive a Cheerio reward. Three readings were taken per animal and averaged. Weight bearing was assessed pre-surgery and 2, 4, 6, 8, 10 and 12 weeks post PMX or sham surgery in male wild-type mice (n = 5/group). One week prior to each testing time point, mice were retrained to the task. Weight bearing was assessed by a blinded observer (NA).

**Quantification of the neuronal signal per region –** All quantifications were performed by an observer blinded to the treatment groups.

Na_V_1.8+ and PGP9.5+ subchondral bone channels were quantified as follows: two sections (80 μm apart) were used to count the number of positive channels. The average number of positive channels was reported as previously described (7). The length of positive channels was measured using ImageJ neuroanatomy plugin (11) as follows: The lengths of all Na_V_1.8+ and PGP9.5+ channels per knee section were measured and the length of the longest channel was reported for that section. The distance from the tidemark was assessed by drawing a straight vertical line between the distal end of Na_V_1.8 and PGP9.5 positive channels and the tidemark (the proximal end being the end connected or close to the bone marrow cavity). This distance was then measured using ImageJ and the shortest distance was reported for each knee section. Since most of the age-matched sham and naïve controls lack these channels, we averaged the distances for all existing Na_V_1.8 and PGP9.5 positive channels in naïve and sham control knees and compared it in all models.

**Knee histopathology –** Knee sections from all groups were stained with hematoxylin (1x-Sigma HHS160) and eosin (1%-Sigma E4382) (H&E). Knee sections were evaluated for cartilage degeneration using a modified OARSI score, as described (7). Four joint surfaces, medial and lateral femoral condyles and tibial plateaux were scored for severity of cartilage degeneration. For each cartilage surface, scores were assigned individually to each of three zones (inner, middle, outer) on a scale of 0–5, with 5 representing the most damage. The maximum score for the sum of 60 femoral and tibial cartilage degeneration on either the medial or lateral side = 30. The maximum possible total cartilage degeneration score for the whole joint (sum of medial and lateral sides) is 60. OARSl scoring was performed twice by an observer blinded to treatment groups and the average of both scores was reported. Synovial hyperplasia, cellularity and fibrosis were evaluated in four joint spaces (lateral femoral, medial femoral, lateral tibial, and medial tibial separately) as described (12). All scores were performed by an observer blinded to treatment groups.

**Supplementary Results**

**Knee histopathology** –We assessed OARSI score, synovitis, and osteophyte width and maturity at different stages of disease.

*OARSI score:* Four and eight weeks after DMM, knees showed mild to moderate cartilage damage (medial cartilage degeneration score for DMM 4+wks = 5.18±1.5, sham 4+wks = 0, DMM 8+wks = 6±1.4, sham 8+wks = 0.2±0.4) (medial cartilage degeneration Suppl. Fig. 3A, and Suppl. Fig. 6F for total cartilage degeneration), Sixteen weeks after DMM, cartilage degeneration was severe (medial cartilage degeneration score for DMM 16+wks = 12.8±3.7), concordant with previous results (7,13,14). Four weeks after PMX, chondropathy was comparable to early DMM, but then progressed to severe cartilage degeneration with full thickness loss in some areas by week 12 (medial cartilage degeneration score for PMX 4+wks = 5.6±1.2, sham 4+wks = 0, PMX 12+wks = 20.6±9.3, sham 12+wks = 0) (medial cartilage degeneration Suppl. Fig. 3D, total cartilage degeneration Suppl. Fig. 6L). One week after ACLR, disease severity was mild 1 week, both in the femur and tibia. By 4 weeks after ACLR injury, severe chondropathy with full thickness cartilage erosion was observed in the femoral condyles, while damage in the tibial plateau was mild (medial cartilage degeneration score for ACLR 1+wk = 3.85±1.8, sham 1+wk = 0.07±0.1, ACLR 4+wks = 16.4±11.2, sham 4+wks = 0) (medial cartilage degeneration Suppl. Fig. 3G, total cartilage degeneration Suppl. Fig. 6R). Naïve 26-week old mice showed no cartilage damage, while by 2 years of age, naïve mice showed mild joint damage in both knee joint compartments (for 26-week old mice: medial cartilage degeneration score = 0.4±0.55 and lateral cartilage degeneration score = 0.98±0.74, medial; for 2-yr-old mice: medial cartilage degeneration score = 4.72±1.19, lateral = 2.08±1.18), (medial cartilage degeneration Suppl. Fig. 3J, total cartilage degeneration Suppl. Fig. 6V), concordant with previously reported findings (12,15). Representative histological images of the medial side are shown in Suppl. Fig. 4. Representative images of the whole joint are shown in Suppl. Fig. 6, in addition to total joint cartilage degeneration scores and total joint synovial scores.

*Synovitis:* In addition to cartilage damage, we also assessed synovial hyperplasia, cellularity, and fibrosis for the four joint quadrants. Four and eight weeks after DMM, synovial changes were observed in the medial femoral and tibial compartments and were significantly more pronounced compared to age-matched shams (Suppl. Fig. 3B shows the total medial synovitis score, Suppl. Fig. 5A-C show the medial hyperplasia, cellularity, and fibrosis scores, Suppl. Fig. 6G shows total synovitis score). Synovitis scores were higher in the 4-week group compared to 8 and 16 weeks (Suppl. Fig. 3B). Significant increase in synovial cellularity was observed 4 weeks after DMM compared to the 8-week and 16-week timepoint (Suppl. Fig. 5B), while no difference was detected in fibrosis and hyperplasia of lining cells between the two timepoints (Suppl. Fig. 5A,C). Similarly, 4 and 12 weeks after PMX, the medial compartment showed pronounced synovitis compared to age-matched shams (Suppl. Fig. 3E shows the total medial synovitis score, Suppl. Fig. 5D-F show the medial hyperplasia, cellularity, and fibrosis scores, Suppl. Fig. 6M shows total synovitis score). The synovial changes peaked 4 weeks after surgery and went down by the 12-week timepoint (Suppl. Fig. 3E). Here too, increased cellularity was observed at 4 weeks compared to 12 weeks after PMX, while a trend of increased hyperplasia and fibrosis was observed at the early timepoint compared to the later timepoint (Suppl. Fig. 5D-F). Thus, findings in both surgical models are concordant with published literature reporting that synovitis scores were highest 4 weeks *post* DMM and reduced with time by week 12 (16).

Synovial changes were also detected 1 and 4 weeks after ACLR, compared to age-matched shams. No significant changes were detected between the 2 timepoints (Suppl. Fig. 3H). shows the total medial synovitis score, Suppl. Fig. 5G-I shows the medial hyperplasia, cellularity, and fibrosis scores, Suppl. Fig. 6S shows total synovitis score). These findings confirm our previous reports of robust synovitis in male mice 1 and 4 weeks after ACLR injury (6).

In naïve mice, 2-year-old mice showed mild medial and lateral synovial changes, a trend of increased cellularity and fibrosis was observed but was not significantly different compared to 26-week-old mice (Suppl. Fig. 3K) and (Suppl. Fig. 5K,L), confirming our recent findings (12). No difference was detected in hyperplasia of lining cells between young and old mice (Suppl. Fig. 4J) and (Suppl. Fig. 6W)

*Osteophytes:* Osteophyte width and maturity were assessed in the medial compartment for each model at both timepoints. DMM operated knees showed medium-sized osteophytes that mature with time over 16 weeks (average osteophyte width at 4 weeks= 183.9±76.6, at 8 weeks= 193.2±69.6, at 16 weeks=220.02± 46.39) (Suppl. Fig 3C). Similarly, four weeks after PMX, medium-sized osteophytes were observed, which further grew into large more mature osteophytes by week 12 (average osteophyte width at 4 weeks= 189.2±56.6, at 12 weeks= 314.7±72.7) (Suppl. Fig 3F). One week after ACLR, knees developed large chondrophytes at both the medial femoral condyle and tibial plateau, further growing and maturing into large, mature osteophytes 4 weeks after injury (average chondrophyte width at 1 week= 250.2±161.3, average osteophyte width at 4 weeks= 336.9.±106.5) (Suppl. Fig 3I). No osteophytes were observed in the lateral compartment of these models. Young naïve mice (26 weeks) did not show osteophytes, while older mice showed chondrophytes/small osteophytes in the medial compartment (average osteophyte width= 138.8±66.8) (Suppl. Fig 3L). Representative histological images of the medial joint are shown in (Suppl. Fig. 4). Representative histological images of whole joint, in addition to total joint cartilage degenerations scores and total joint synovial scores are shown in (Suppl. Fig. 6).

Representative histological images of the medial side are shown in Suppl. Fig.4. Representative images of the whole joint are shown in Suppl. Fig. 6, in addition to total joint cartilage degeneration scores and total joint synovial scores.

**Knee hyperalgesia** ­– PMX mice developed knee hyperalgesia 4 and 8 weeks after surgery compared to sham-operated mice and age-matched naïve mice (Suppl. Fig. 10A).

**Weight bearing asymmetry** ­– Mice developed weight bearing asymmetry starting at 10 weeks after PMX surgery and continues up to 12 weeks (Suppl. Fig. 10B).

**References**

1. Stirling CL, Forlani G, Baker MD, Wood JN, Matthews EA, Dickenson AH, et al. Nociceptor-specific gene deletion using heterozygous NaV1.8-Cre recombinase mice. Pain. 2005 Jan;113(1):27–36.

2. Shields SD, Ahn HS, Yang Y, Han C, Seal RP, Wood JN, et al. Nav1.8 expression is not restricted to nociceptors in mouse peripheral nervous system. Pain. 2012 Oct;153(10):2017–30.

3. Glasson SS, Blanchet TJ, Morris EA. The surgical destabilization of the medial meniscus (DMM) model of osteoarthritis in the 129/SvEv mouse. Osteoarthritis Cartilage. 2007 Sep;15(9):1061–9.

4. Knights CB, Gentry C, Bevan S. Partial medial meniscectomy produces osteoarthritis pain-related behaviour in female C57BL/6 mice. Pain. 2012 Feb;153(2):281–92.

5. Rzeczycki P, Rasner C, Lammlin L, Junginger L, Goldman S, Bergman R, et al. Cannabinoid receptor type 2 is upregulated in synovium following joint injury and mediates anti-inflammatory effects in synovial fibroblasts and macrophages. Osteoarthritis Cartilage. 2021 Dec;29(12):1720–31.

6. Bergman RF, Lammlin L, Junginger L, Farrell E, Goldman S, Darcy R, et al. Sexual dimorphism of the synovial transcriptome underpins greater PTOA disease severity in male mice following joint injury [Internet]. Physiology; 2022 Dec [cited 2023 Jan 26]. Available from: http://biorxiv.org/lookup/doi/10.1101/2022.11.30.517736

7. Obeidat AM, Miller RE, Miller RJ, Malfait AM. The nociceptive innervation of the normal and osteoarthritic mouse knee. Osteoarthritis Cartilage. 2019 Nov;27(11):1669–79.

8. Miller RE, Ishihara S, Bhattacharyya B, Delaney A, Menichella DM, Miller RJ, et al. Chemogenetic Inhibition of Pain Neurons in a Mouse Model of Osteoarthritis. Arthritis Rheumatol Hoboken NJ. 2017 Jul;69(7):1429–39.

9. Ishihara S, Obeidat AM, Wokosin DL, Ren D, Miller RJ, Malfait AM, et al. The role of intra-articular neuronal CCR2 receptors in knee joint pain associated with experimental osteoarthritis in mice. Arthritis Res Ther. 2021 Apr 7;23(1):103.

10. Blackwell AA, Banovetz MT, Qandeel, Whishaw IQ, Wallace DG. The structure of arm and hand movements in a spontaneous and food rewarded on-line string-pulling task by the mouse. Behav Brain Res. 2018 Jun;345:49–58.

11. Arshadi C, Günther U, Eddison M, Harrington KIS, Ferreira TA. SNT: a unifying toolbox for quantification of neuronal anatomy. Nat Methods. 2021 Apr;18(4):374–7.

12. Geraghty T, Obeidat AM, Ishihara S, Wood MJ, Li J, Lopes EBP, et al. Age‐associated changes in knee osteoarthritis, pain‐related behaviors, and dorsal root ganglia immunophenotyping of male and female mice. Arthritis Rheumatol. 2023 Apr 25;art.42530.

13. Little CB, Barai A, Burkhardt D, Smith SM, Fosang AJ, Werb Z, et al. Matrix metalloproteinase 13-deficient mice are resistant to osteoarthritic cartilage erosion but not chondrocyte hypertrophy or osteophyte development. Arthritis Rheum. 2009 Dec;60(12):3723–33.

14. Miller RE, Tran PB, Ishihara S, Larkin J, Malfait AM. Therapeutic effects of an anti-ADAMTS-5 antibody on joint damage and mechanical allodynia in a murine model of osteoarthritis. Osteoarthritis Cartilage. 2016 Feb;24(2):299–306.

15. Loeser RF. Aging processes and the development of osteoarthritis. Curr Opin Rheumatol. 2013 Jan;25(1):108–13.

16. Shu CC, Zaki S, Ravi V, Schiavinato A, Smith MM, Little CB. The relationship between synovial inflammation, structural pathology, and pain in post-traumatic osteoarthritis: differential effect of stem cell and hyaluronan treatment. Arthritis Res Ther. 2020 Feb 14;22(1):29.
